# *In situ* analysis of titanium isotope ratios in stardust using LA-CC-MC-ICPMS/MS†

**DOI:** 10.1039/d5ja00068h

**Published:** 2025-05-15

**Authors:** Kathryn M. M. Shaw, Markus Pfeifer, Benjamin L. L. Coath, Jamie Lewis, Dan Bevan, Christopher D. Coath, Tim Elliott

**Affiliations:** a School of Earth Science, University of Bristol Queens Road Bristol BS8 1RJ UK Kathryn.shaw@bristol.ac.uk; b Thermo-Fisher Scientific (Bremen) GmbH Hanna-Kunath St. 11 28199 Bremen Germany; c Centre for Exploration Targeting, School of Earth Sciences, University of Western Australia Perth Western Australia Australia

## Abstract

Presolar grains are nanometre-scale dust grains that exhibit large isotope excursions that illustrate the stellar isotopic input into the Solar System. Further, it is thought that they were differentially incorporated into meteorite parent bodies and thus can be used to trace planetary genetics and construction. *In situ* mapping of the distribution of presolar grains in the matrix of primitive meteorites therefore provides a key means to achieve this goal. However, *in situ* methods complicate isotopic measurements, such as those of Ti, due to their large isobaric interferences. To enable such measurements a prototype a collision cell, multicollector inductively-coupled plasma mass spectrometer with a pre-cell mass filter (CC-MC-ICPMS/MS) was developed and called Proteus. In this study we show that, when coupled to a laser ablation system, Proteus has the capability to measure, *in situ*, large Ti isotope excursions such as those expected in presolar grains (>200‰). Within the collision cell we introduced O_2_ gas and react Ti^+^ to TiO^+^ and perform the multi-collector isotope ratio measurement on the TiO^+^ species. The presence of isobaric interferences from Ca^+^, V^+^, and Cr^+^ are greatly reduced due to their lower ion reaction efficiency with O_2_ gas. The measurement of TiO^+^ using the pre-cell mass filter ensures that these ions are measured in a cleared region of the mass-spectrum where a Ni^+^, Cu^+^, and Zn^+^ ions would otherwise be present as interferences. Using this technique, complex rock samples with high Ca/Ti and Cr/Ti, for example BIR-1G, give the same mass-independent isotopic Ti ratios as essentially pure Ti-minerals, *e.g.* brookite. By reducing isobaric interferences from *in situ* measurements we can detect the large isotopic excursions in presolar grains without the added impediment of non-solar interference corrections for isobaric interferences.

## Abstract 2

*In situ*, high spatial resolution, laser-ablation CC-MC-ICPMS/MS method for presolar Ti isotopes using O_2_ gas to reduce isobaric interferences.

## Graphical abstract

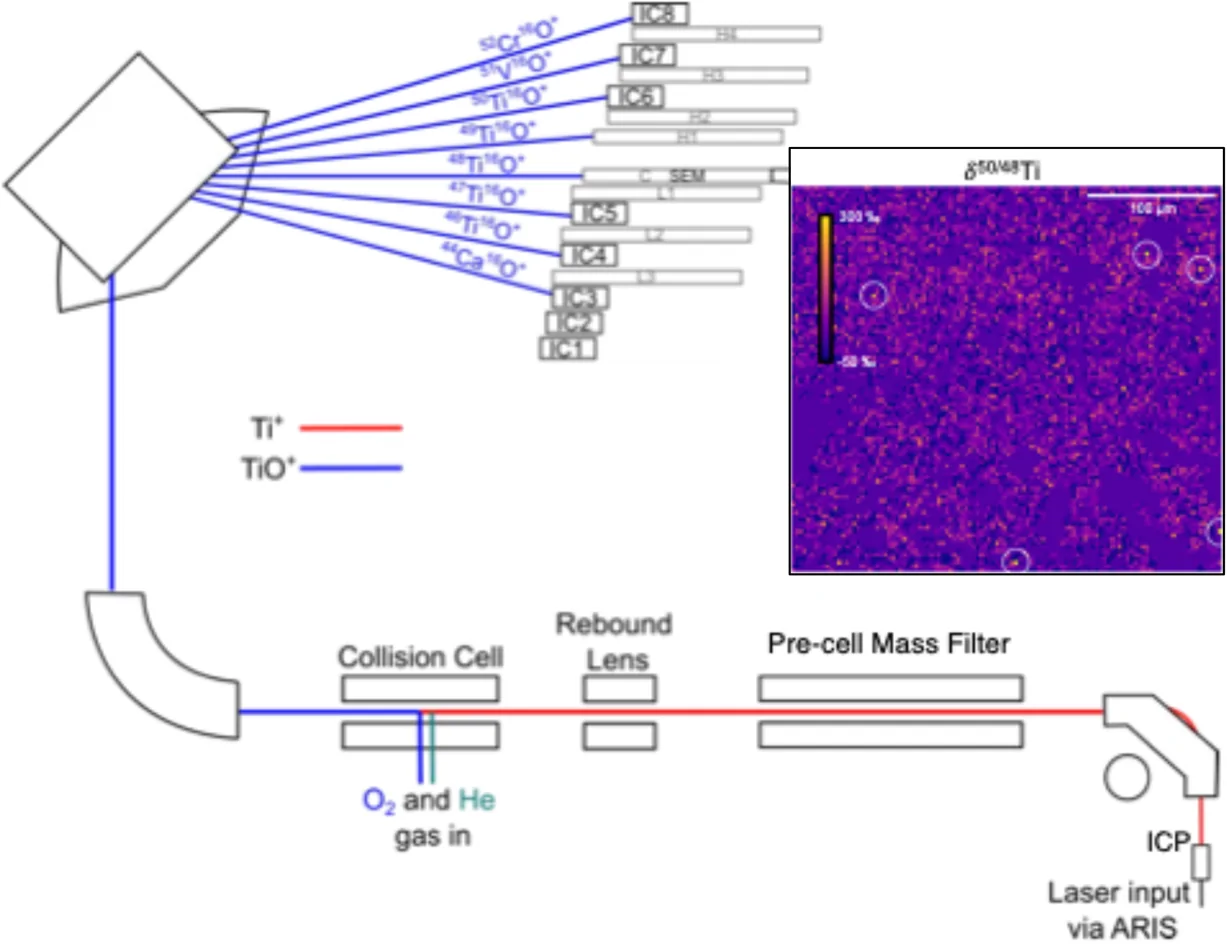

## 1 Introduction

The Solar System is a blend of material originating from different stellar sources.^1^ Considerable isotopic excursions (>100‰) measured in individual, sub-micrometre grains within the matrices of unequilibrated chondrites have led to widespread agreement of their presolar origin.^2^ Their distinct isotopic signatures provide a direct line of evidence for processes occurring during stellar nucleosynthesis and are used to ground hypotheses from models and spectral data.^3–5^ Further evidence for the presence of presolar grains comes from bulk meteorite analyses that display small but significant differences in their mass-independent isotopic compositions of various elements.^6^ Some studies have suggested a variable distribution of previously identified presolar grains to explain certain mass-independent isotopic differences between bulk meteorite compositions,^7^ such as presolar SiC grains for s-process Mo isotopes.^8,9^ However, no presolar grain found to date is evidently responsible for the highly distinctive, coupled variations in ^46^Ti and ^50^Ti observed between bulk meteorite analyses.^10^ Given these well-defined isotopic differences found in the Solar System for Ti,^10^ determining the nature and distribution of the presolar Ti carrier could help clarify how the Solar System formed distinct reservoirs of presolar material, *e.g.* through large-scale nebula heterogeneities or unmixing processes driven by size or thermal susceptibility.^10–14^

Previous measurements of Ti in presolar grains using NanoSIMS (Nanoscale Secondary Ion Mass Spectrometry) have found isotopic compositions (*δ*^50/48^Ti > 200‰) mainly associated with formation in red giants, such as those in presolar SiC grains, with a few presolar oxides exhibiting compositions (*δ*^50/48^Ti > 1000‰) more suggestive of supernovae processes.^15–19^ However, none of these previously identified grains carry the appropriate Ti signatures to conclusively explain the bulk meteorite isotopic variations.^10^ A key issue is that current studies only measure Ti in presolar grains found in size-filtered batches from the residues of harsh chemical dissolutions of primitive meteorites. Given some oxide and all silicate presolar grains are likely destroyed during such traditional isolation methods, these studies may therefore not provide a representative sample of all presolar grains. Indeed, presolar silicate grains are second only to presolar nano-diamond grains in their abundance in primitive meteorites, suggesting they could be of significant importance.^20^ An *in situ* method is required for the full presolar Ti inventory to be characterised. Some *ex situ* data do exist indicating the possible presence of ^50^Ti-rich grains inferred from enrichments at mass-50 (ref. 21) but as both ^50^Cr and ^50^Ti contribute to the measured signal as mass-50, there is some ambiguity about the exact nature of the anomaly. Indeed, correction of isobaric interferences is a major problem for *in situ* analyses of Ti isotope ratios. Given the isotopic ratios of interfering elements are unknown for presolar samples the accuracy of traditional interference corrections is compromised, *i.e.* monitoring other isotopes of the interfering element and assuming terrestrial isotope ratios. Thus, NanoSIMS techniques for the identification of sub-micron sized presolar grains by *in situ* isotopic mapping of elements have currently been limited to elements of sufficiently high abundance in presolar grains with minor or no isobaric interferences (*e.g.* O, Si, Mg).^22^ As a result of the challenging and abundant elemental isobaric interferences on the Ti mass spectrum, which will be contributed from the presolar grains and the surrounding matrix, no data exist yet for *in situ* presolar Ti isotope ratios. A novel *in situ* technique which removes the isobaric interferences before isotope collection would allow an accurate view to be gained of the Ti presolar grain inventory.

In this study, we explore the capacity for *in situ* measurement of Ti isotope ratios in presolar grains within primitive chondrite matrices using laser ablation coupled to Proteus, a proto-type collision cell, multicollector inductively-coupled plasma mass spectrometer with a pre-cell mass filter, LA-CC-MC-ICPMS/MS. As stressed above, the isotopic ratios of the interfering elements from presolar grains are unknown and so the magnitude of the interference must be minimised for the most reliable determinations. We use the collision cell in combination with a pre-cell mass filter on Proteus to remove or reduce unresolvable interfering species, which come from the busy measured spectrum of *in situ* analyses^23^ and the argon plasma itself.^24^ Titanium displays a differential reactivity with O_2_ in the collision cell compared to its isobaric interferences Ca, V, and Cr. Thus, by measuring Ti isotopes as their oxide adducts, *e.g.*, ^46–50^Ti^16^O^+^, we can greatly reduce elemental isobaric interferences, which do not form oxides as efficiently as Ti. The pre-cell mass filter is set to limit the ions produced in the plasma to a selected mass/charge (*u*/*q*) range, *e.g.* 43–53 *u*/*q*, so that only the ions of interest progress into the collision cell. This ensures that the freshly-generated TiO^+^ ions are measured in the cleared mass range of 62–66 *u*/*q*.

## 2 Methods

### 2.1 Sample preparation

Prior to the *in situ* work, some instrumental calibration is undertaken using solution analyses. A reference Ti solution was prepared from batch no. 992801 of standard reference material (SRM) 3162a from the National Institute of Standards and Technology (NIST). A mixed solution was prepared with 0.2 ng per ml Ca, 0.01 ng per ml Ti, 0.001 ng per ml V, 0.05 ng per ml Cr, such that its elemental ratios are similar to those found in carbonaceous chondrites, the target samples for this method. This solution was made from NIST SRM 3621a for Ti and Atomic Standard pure element solutions for Ca, V and Cr.

In setting up *in situ* analyses, we used glass reference materials including basalt BIR-1G from the United States Geological Survey (USGS) and NIST glass SRM 610. These were mounted into a 1-inch round in resin and then cut and polished down to a 100 nm grade. Additional in-house minerals are also used as external standards and prepared in a similar way to the glass reference materials above. These consisted of brookite (TiO_2_), titanite (CaTiSiO_5_), wollastonite (CaSiO_3_) and hibonite (CaAl_12_O_19_), and were found to be of sufficient purity and homogeneity from previous microprobe analysis. Reference concentrations of elements in BIR-1G and NIST-610 (ESI Table 1†) were based on previous published concentrations in the GEOREM database.^25^

Isotopic analyses by laser ablation for samples and reference materials are externally normalised to a pressed tablet of nano powdered meteorite. The parent material for this pressed tablet is an ordinary H4 chondrite (privately acquired and informally labelled M12), which has a sufficiently comparable matrix to the samples, and due to its metamorphic grade should lack presolar grains. We subsequently refer to this standard as H4-OC. In preparing this sample, 2 g of the ordinary chondrite was powdered in a ball-mill. The metal flakes were then removed *via* a sieve to aid in ablation characteristics. The powder was then compressed into a tablet on a pneumatic press with pressure applied for 1 minute. Reducing the metal abundance has little effect on the Ti concentration of the powder, which still had similar Cr/Ti, Ca/Ti, and V/Ti to the sample meteorites (ESI Table 1†).

### 2.2 Sample introduction

The initial setup of the Ti method in each session is undertaken with standard solutions since solution aspiration typically makes for a steadier ion beam from the plasma source than laser ablation. The setup consists of instrumental tuning, Faraday cup and ion counter calibration, and the tuning of gas flows to reduce the monitored molecular interference intensities (*i.e.*, Cr^16^O^+^, Section 2.3). Solution work is carried out with a dry plasma using an Aridus II desolvating nebuliser with Ar sweep gas and additional N_2_ gas for increased sensitivity. We use a standard Thermo Scientific® iCAP-Q sampler cone and cold-plasma skimmer cone, which increases sensitivity by a factor of three with only a slight increase in Ar-based interferences compared to the regular iCAP-Q skimmer cone with insert. Samples are introduced in a 0.3 M HNO_3_ solution. The lower extraction of −300 V on Proteus, compared to −2000 V on a Thermo Scientific® Neptune Plus MC-ICPMS, results in a Ti sensitivity a factor of four lower than the Neptune.

Laser ablation is undertaken using a Photon-machines® Analyte G2 193 nm excimer laser with a two-volume HelEx II® cell. Optimal sample transfer from the laser to the mass spectrometer is achieved with He flow rates of 0.450 and 0.350 L min^−1^ to cell and cup respectively, in the two-volume system. The movement of the sample stage in *X* and *Y* is controlled with two stepper motors. Focusing in *Z* is achieved by controlling the objective lens assembly *via* a third stepper motor. The movement of the *Z*-axis is important during analysis to achieve optimal focus of the laser on the sample at small (typically 1–3 μm) spot sizes. Due to the lack of temperature regulation of the laser stage and heat fluctuations associated with laser use, the *Z*-axis can shift by > 10 μm over hour timescales. The shift is sufficient to defocus the laser beam at small spot sizes and reduce the ablation yield and, therefore, overall measured sensitivity. Thus, after prolonged use of the laser, the *Z*-axis often requires refocusing to maximise ablation conditions again. Our method uses a TeledyneCetac® ARIS (Aerosol Rapid Introduction System) which consists of a short tubing length with an inside diameter of ∼1 mm which is retrofitted into the laser cell and connects directly to the plasma torch. The ARIS reduces the time taken for the ablated aerosols to travel from the sample to the plasma to approximately 0.1 s, compared to the longer transit times of conventional aerosol transfer systems.

During analysis, two main laser ablation patterns were used. First, data on homogeneous standard materials were collected using rasters with a scan speed of 10 μm s^−1^, spot sizes from 1–5 μm, and repetition rates of 4 or 8 Hz. Second, meteorite sample mapping was achieved by line transects across a sample in the *X*-direction from left to right, repeated after an incremental off-set in the *Y*-direction. Rasters were not used for spatial mapping due to the inability to distinguish where one line ended and another began in the collected data. To avoid this problem in the line scan mode, a time gap with no ablation of 5 s between laser lines was used to identify where each line began and ended in each dataset. The pixel size in the *Y*-direction on the maps is defined by the laser spot diameter, which is usually 2 or 3 μm. As we used continuous line scans, the laser scan speed and mass-spectrometer integration time define the pixel size in the *X*-direction, assuming the washout is shorter than the integration time. We best attempted to match laser repetition rates with possible instrumental integration times, permitted by the mass-spectrometer software. This resulted in using a 0.131 s integration time with an 8 Hz laser repetition rate, which produces one laser pulse every 0.125 s. The sample scan speed was then set to best match the spot resolution *i.e.*, for a 3 μm spot, with an integration time of 0.131 s, the scan speed is set to approximately 23 μm s^−1^ which equates to a travel of ∼3 μm between integrations. The approach minimises ablation depth, *i.e.*, there is only one laser pulse per measurement per spot area, which maximises the volume resolution in the *Z*-direction. The ablation depth ranged between 0.4 and 0.8 μm on the meteorite samples. The ablation parameters are shown in ESI Table 2.†

In keeping with the two different modes of data collection, we report data from homogeneous standards and samples in two different ways. For the standards, we average all the individual integrations in one laser raster, whereas for the samples, we treat each individual integration as a single data point to construct an isotopic map. We use the term ‘pixel’ to refer to such a measurement of a single integration from a sample.

### 2.3 CC-MC-ICPMS/MS setup

**Fig. 1:**
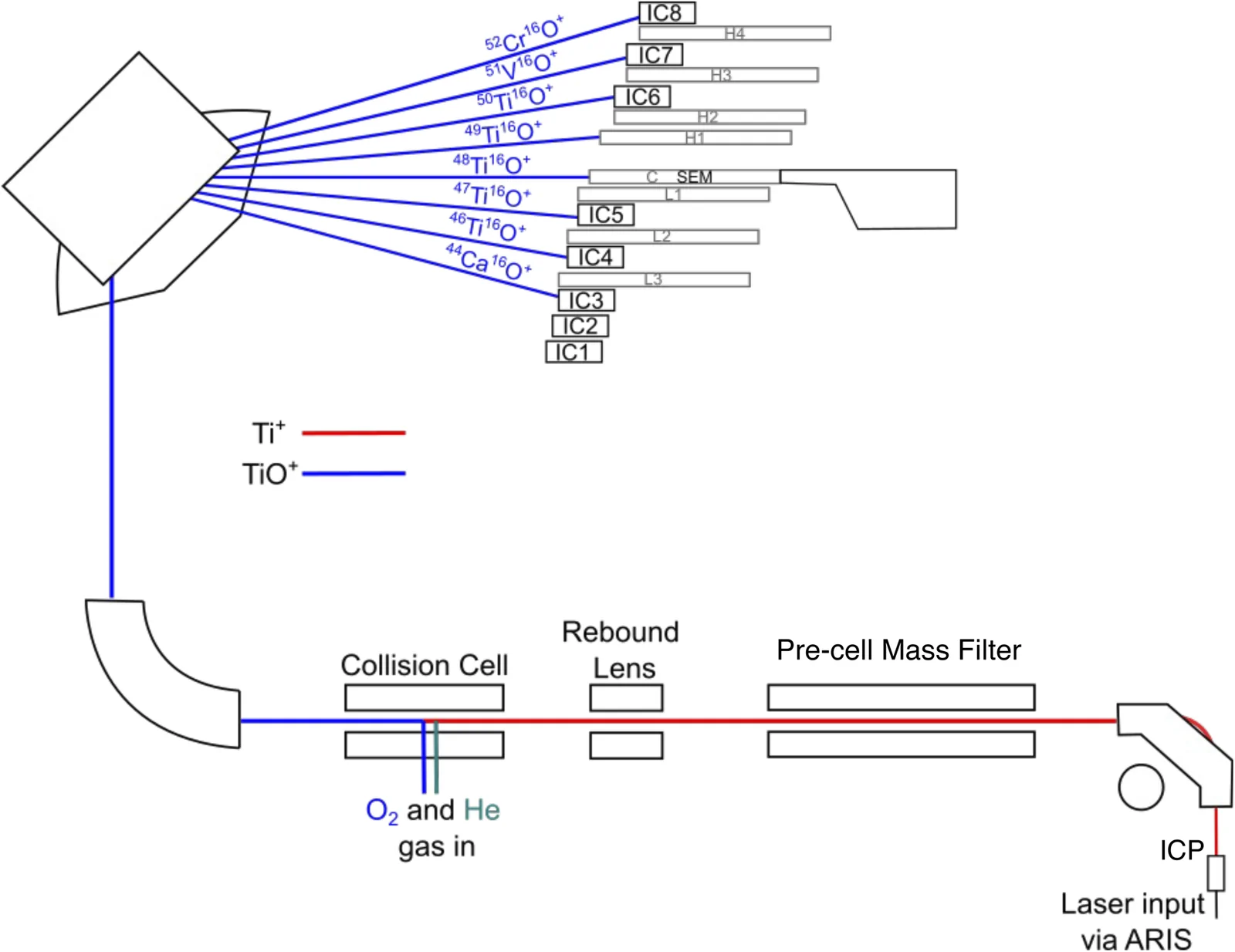
Schematic of Proteus CC-MC-ICPMS/MS. The detectors used to collect the ion beams of interest are indicated. It is impossible to measure all beams of interest on ion counting devices (IC or SEM), so ^49^Ti^16^O^+^ enters Faraday cup (H1). In one variant configuration, with modified relative collector positions, ^49^Ti^16^O^+^ was collected on the central SEM and ^48^Ti^16^O^+^ on IC5 leaving ^47^Ti^16^O^+^ un-collected.

**Fig. 2:**
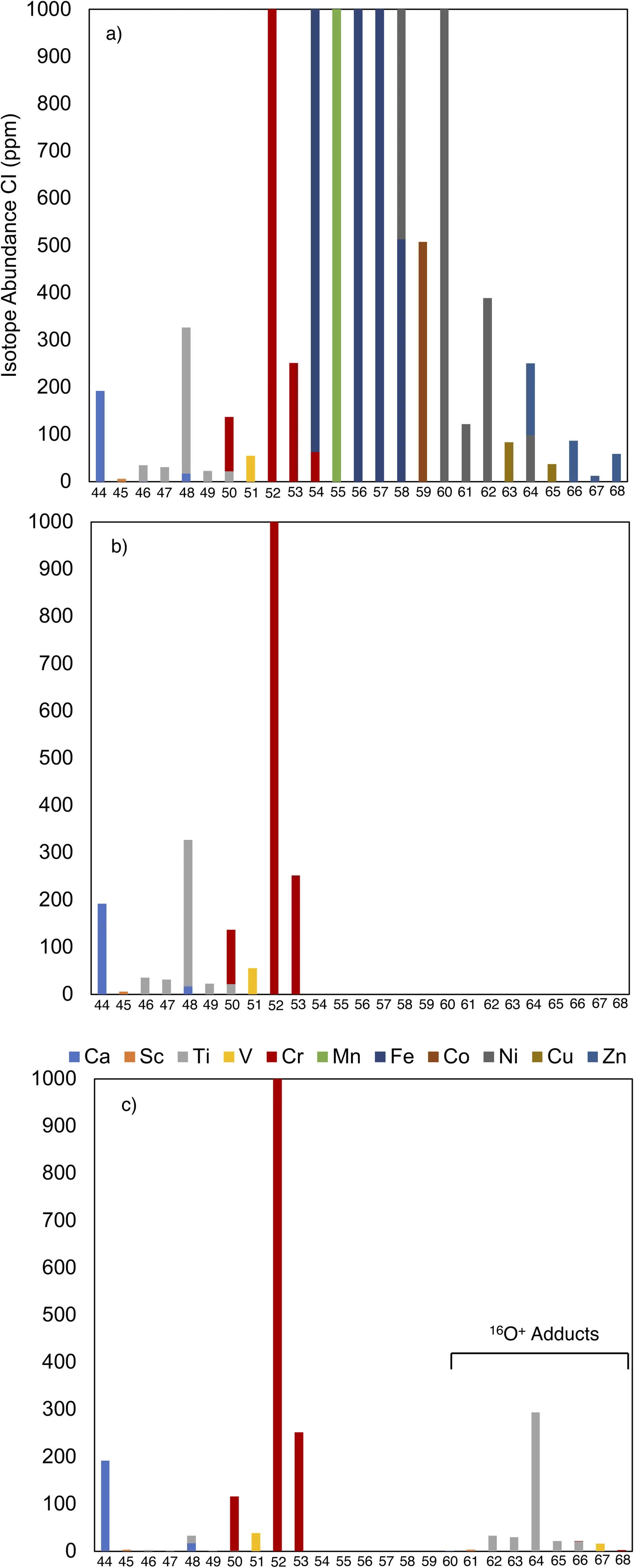
Illustrative figure of how the ‘mass shift’ method works to reduce isobaric interferences on the measured Ti spectrum, (a) isotopic abundances of a CI chondrite before operation of collision cell or pre-cell mass filter; (b) after the pre-cell mass filter with mass transmission (assumed 100%) only in a window of 48 ± 5 *u*/*q*; (c) after the pre-cell mass filter and collision cell operated with oxygen assuming typical Ti, Ca, V, and Cr oxide ion production efficiencies determined by our measurements. Note that in (c) the mass shifted Ca, Sc, Ti, V, and Cr species are shown as ^16^O oxide adducts only. Relative intensities of Ti ion species relative to Ca, V and Cr interferences in the oxide spectrum is representative of what we achieve using Proteus during this study.

Ions from the plasma first pass through a pre-cell quadrupole mass filter (Fig. 1). For this study, the mass filter operates in ‘window’ transmission mode. A window ∼10 *u*/*q* wide, centred on mass 48 is used, to allow transmission of ^44^Ca^+^, ^46–50^Ti^+^, ^51^V^+^ and ^52^Cr^+^ (Fig. 2b and ESI Fig. 1†). The range is chosen to prevent any transmission of Ar^+^ or ArO^+^ into the collision cell which otherwise results in Ar-based molecular interferences and reactions that degrade the oxide reaction efficiency. Given that we measure Ti as an oxide in the range of 62 to 66 *u*/*q* (see below), the pre-cell mass filter stops the direct transmission of ions in this mass range such as Ni^+^, Cu^+^ and Zn^+^. These elements are particularly abundant in chondritic meteorites, relative to most terrestrial samples, increasing the importance of their exclusion. Thus, the pre-cell mass filter ensures that newly created ^*x*^Ti^16^O^+^ species are measured against very low backgrounds (Fig. 2b and c).

Proteus' pre-cell quadrupole mass filter differs from the double Wien approach used in the commercially available Thermo Scientific® Neoma CC-MC-ICPMS/MS. The Thermo Scientific® iCAP-Q quadrupole filter in Proteus is able to create a mass window with transmission as narrow as a single *u*/*q* with relatively sharp mass cut-off *i.e.* 100% to 0% transmission over 1 *u*/*q*. In contrast, the double Wien approach, with central “selectable” slit, on the Neoma MS/MS is limited with a less sharp mass cut-off, which results in the inadvertent trimming of the desired masses when trying to fully exclude the Ar^+^ and ArO^+^.^26^ Thus, we were unable to fully stop Ar^+^ and ArO^+^ from entering the collision cell while testing the transmission of the Ti isotope spectrum on the Neoma MS/MS at the University of Bristol.

In this application, the rebound lens (see Fig. 1) is not set to deflect the ion beam, so ions pass straight through into the collision cell. The collision (or reaction) cell is a sub-volume of the vacuum system that can be pressurised with collision or reaction gases. The cell in Proteus contains a quadrupole with true hyperbolic geometry rods, with an applied (RF only) voltage, which acts as an ion guide. It is also equipped with an axial field^27^ by means of DC voltages applied to a series of electrodes along the length of the cell. This axial field can be adjusted to subtly accelerate or decelerate the ions as they pass through the cell and interact with the gas molecules. Ion-molecule reaction cross sections are sensitive to the relative velocities of the reactants; hence, the axial field can be used to inhibit or enhance certain reactions. A slightly negative potential difference, totalling ∼ −20 V across the electrodes, accelerates ions through the collision cell and is found to limit the production of undesirable polyatomic species (*e.g.*, ArO_2_^+^, ArCO^+^, O_4_^+^), while maintaining good TiO^+^ intensity. Approximately −10 V is applied to the collision cell exit lens to focus the ions into the mass spectrometer.

The Proteus collision cell is supplied with two independently controlled gas flows: (1) a mixture of 0.5% O_2_ (99.999% purity) in He gas (99.9999% purity) and (2) He gas (purity 99.999%). High purity gases were chosen in order to limit possible impurity-driven side reactions in the collision cell which may give rise to additional interferences. The gases are also passed through gas-specific purifiers on the path to the collision cell to further increase their purity. With flows between 2.5–3.5 ml min^−1^ and 3.0–4.0 ml min^−1^ of O_2_–He mix and He, respectively, Ti^+^ ions react efficiently to produce their TiO^+^ adducts. The reactivity of O_2_ with Ti^+^ relative to Cr^+^ and V^+^ is strongly dependent on the amount of O_2_ available in the collision cell. The TiO^+^ yield increases with increasing O_2_ addition until ∼85% of the Ti^+^ is oxidised. Further increasing the O_2_ has little benefit but, rather, the interfering species CrO^+^ and VO^+^ increase in intensity. Hence, the flow rate of O_2_ is tuned to maximise TiO^+^ formation without increasing CrO^+^ or VO^+^ to limit the interference on any Ti isotope intensity to <10‰ (ESI Fig. 2†). The addition of pure He increases the yield of TiO^+^ through collisional focussing of the ion beam in the cell.^28^

**Fig. 3:**
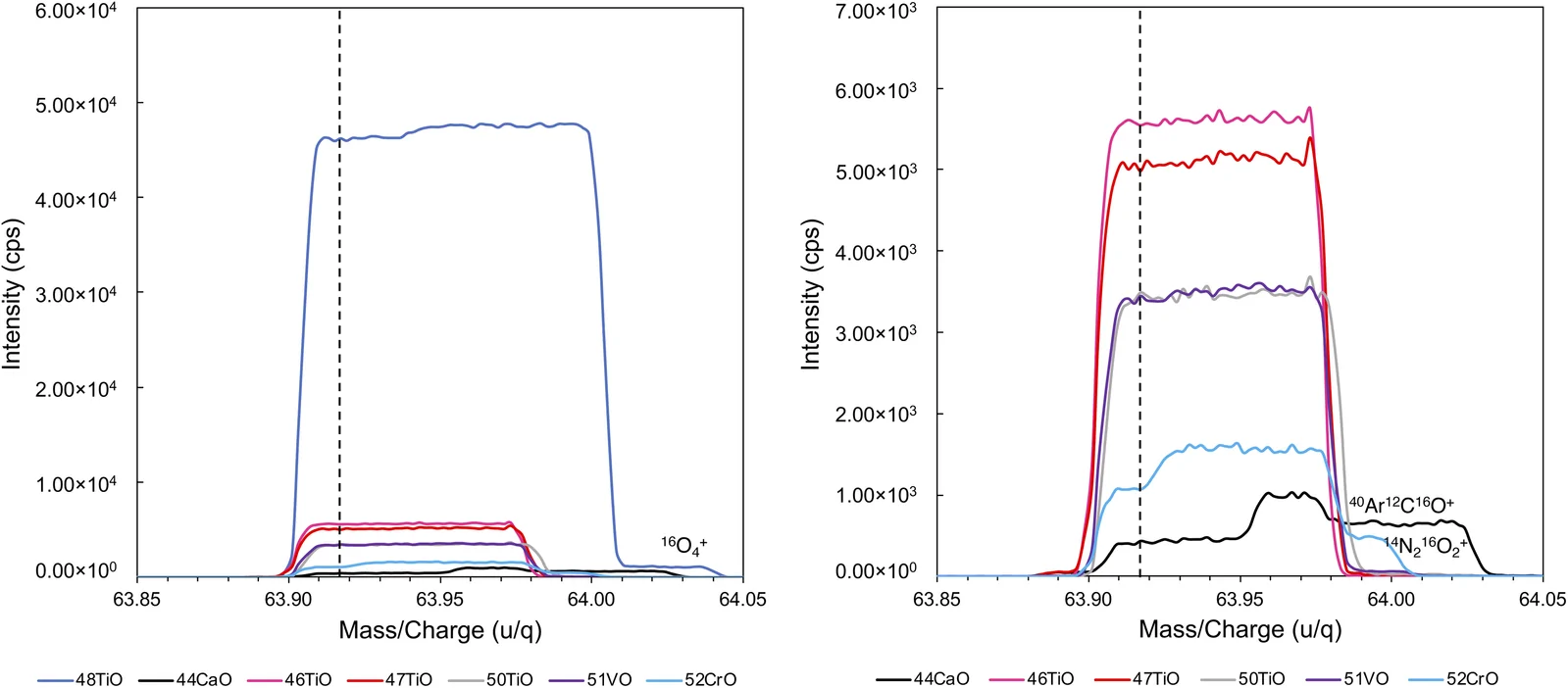
Mass scans of TiO^+^ species and isobaric interferences measured on ion-counting detectors. Dashed black line represents the collection mass where we avoid molecular interferences by sitting on the resolved “shoulder” of TiO^+^ beams. Right shows the same scans as left but with an expanded scale and without the ^48^TiO^+^ beam to highlight the ‘shoulders’ on the smaller signals.

We use a ‘medium resolution’ source slit which yields a mass resolution of >6500 *M*/Δ*M* (5–95% peak edge definition). This is sufficient to resolve key molecular interferences such as ^16^O_4_^+^ on ^48^Ti^16^O^+^ (0.037 *u*/*q* offset), ^14^N_2_^16^O_2_^+^ on ^44^Ca^16^O^+^ (0.046 *u*/*q* offset), and ^36^Ar^16^O_2_^+^ and ^40^Ar^12^C^16^O^+^ on ^52^Cr^16^O^+^ (0.022 *u*/*q* offset) (Fig. 3).

### 2.4 Multi-ion detection and cup configuration

The TiO^+^, CaO^+^, VO^+^, and CrO^+^ ion species are measured on Compact Discrete Dynode (CDD) ion counters, mounted on Faraday carriages (H4, H3, H2, L1, L2, L3), or on a fixed, secondary-electron multiplier (SEM) at the centre cup position (Fig. 1). Such ion-counting devices provide low detection limits that are key to measuring individual presolar grains containing, typically, only fg of Ti. Proteus has insufficient ion counters for all species of interest to be measured simultaneously; we tried measuring ^49^Ti^16^O^+^ using a Faraday cup attached to an amplifier equipped with a 10^13^ Ω feedback resistor. However, owing to the highly transient laser signals, coupled with the slow amplifier response and decay time, this proved unsuccessful for this application. Instead, a second cup configuration was designed to monitor ^49^Ti^16^O^+^ at the expense of ^47^Ti^16^O^+^ (Table 1). Both these configurations were used to collect data on presolar grains for the full suite of Ti isotopes, albeit in different sessions.

Cup configurations used in this study on a bespoke multi-ion counting array for Proteus

**Table:** 

Config. 1	IC3	IC4	IC5	IC1/SEM	H1	IC6	IC7	IC8
	^44^Ca^16^O^+^ (L3)	^46^Ti^16^O^+^ (L2)	^47^Ti^16^O^+^ (L3)	^48^Ti^16^O^+^ (C)	^49^Ti^16^O^+^	^50^Ti^16^O^+^ (H2)	^51^V^16^O^+^ (H3)	^52^Cr^16^O^+^ (H4)

**Table:** 

Config. 2	IC3	IC4	IC5	IC1/SEM	H1	IC6	IC7	IC8
	^44^Ca^16^O^+^ (L3)	^46^Ti^16^O^+^ (L2)	^48^Ti^16^O^+^ (L3)	^49^Ti^16^O^+^ (C)	—	^50^Ti^16^O^+^ (H2)	^51^V^16^O^+^ (H3)	^52^Cr^16^O^+^ (H4)

For measurements of Ti in solution, where the monoatomic ion species are analysed, the static collection of ^44^Ca^+^ and ^52^Cr^+^ usually requires a dynamic method. When measured as oxides, the masses of the measured species are greater, decreasing their relative mass differences and allowing their simultaneous collection using a static method.

### 2.5 Laser ablation routine

A typical session routine was set up as follows. The wollastonite standard was analysed as the first and last measurements in the entire session to determine Ca-related interference corrections, as described below. A suite of standards including brookite, titanite, hibonite, BIR-1G, NIST-610 are run as a block of measurements at the start and end of each session to assess the quality of data across a range of chemical and material matrices. The sample meteorite is mapped as four separate rectangular sections of 25 or 30 lines of approximately 300 μm or 250 μm in length. Each section contains a quarter of the total number of lines which make up a map of approximately 100 or 120 lines. Maps with 3 μm spot resolution contained 100 lines at 300 μm length so a total area of approximately 90 000 μm^2^. Maps with 2 μm spot resolution contained 120 lines at 250 μm length so a total area of approximately 60 000 μm^2^. The measurement of each quarter of the sample or a block of standards is bracketed with measurements of the H4-OC nano-powder standard, which is thus analysed around every 5–15 minutes. Such a session takes 2–3 hours in total.

All measurements used a 0.131 s integration time, a pre-set value permitted by the instrument control software (see Section 2.2). Measurements of homogenous standards, consisting of 1000–2000 integrations, are reported as the mean and SE of all the integrations collected during ablation. For sample maps, each integration is treated as an individual measurement and these integrations are then reported as individual pixels on a sample map.

### 2.6 Offline corrections

All data processing is undertaken offline using a purpose-built code in Python.

#### 2.6.1 Blank and background signal subtraction

The background signal is quantified by collecting data for 1 minute, without ablation, directly before and after a period of ablation for standard and sample data collection (*e.g.* between 5 and 15 minutes respectively). The mean of these two ‘blank’ measurements is then subtracted from each integration collected during the intervening ablation. The background intensity is usually less than 5% of the ablation signal.

#### 2.6.2 Ca-related interferences

Calcium-related interferences were corrected using analyses of an in-house wollastonite standard (with negligible Ti, V, and Cr abundances) at the start, middle, and end of each session (1–3 hours). Interferences such as ^46,48^Ca^16,17,18^O^+^ and ^44,46,48^Ca^19^F^+^ were found to occur on all peaks of interest other than mass 68 (^52^Cr^16^O^+^). Therefore, the intensities during wollastonite ablation are measured on all collectors. The Ca-related interferences are ratioed to the ^44^Ca^16^O^+^ signal to determine relative production factors. The factors are used with the measured ^44^Ca^16^O^+^ signals collected during standard and sample measurements to correct for any Ca-related interferences on the TiO^+^ peaks. This approach monitors possible session to session variations in CaO^+^ and CaF^+^ formation in the collision cell as a result of residual gases from other users (*e.g.* SF_6_). However, Ca-related corrections are minimal (≪1% of Ti signals).

#### 2.6.3 Minor oxygen isotope correction

The influence of adducts with the minor oxygen isotopes were corrected for using the ^17^O/^16^O and ^18^O/^16^O ratios of the gas as determined by single mass transmission of ^*x*^Ti^+^ ions through the quadrupole pre-cell mass filter allowing direct collection of un-interfered ^*x*^Ti^16,17,18^O^+^ signals from a pure Ti solution (ESI†). The pure ^*x*^Ti^16^O^+^, ^*x*^Ti^17^O^+^, ^*x*^Ti^18^O^+^ signals were then ratioed to give the relative formation efficiency of the minor TiO species. These effective ^17^O/^16^O and ^18^O/^16^O gas ratios were used in progressive subtractions for minor oxide contributions to beam intensities of increasing *u*/*q* starting from ^46^Ti^16^O^+^. For example, multiplying the observed ^46^Ti^16^O^+^ by the determined ^17^O/^16^O and ^18^O/^16^O ratios, gave signal intensities of ^46^Ti^17^O^+^ and ^46^Ti^18^O^+^ which were subtracted from ^47^Ti^16^O^+^ and ^48^Ti^16^O^+^ respectively. The now minor oxide corrected ^47^Ti^16^O^+^ is then used to calculate ^47^Ti^17^O^+^ and ^47^Ti^18^O^+^ to be subtracted and so on.

#### 2.6.4 Mass bias

Owing to the disparity in the nature of materials and consequent style of data collection between standards and samples (Section 2.5), we reduce and report the data from standards and sample maps differently.

For samples, mass bias is corrected externally by normalising sample pixels to the mean ratios of the bracketing standard, namely our in-house mount of nano-powdered H4-OC. We note that while the latter likely has a slightly distinct Ti isotope ratio to terrestrial values (*e.g.*, Trinquier *et al.*, 2009),^10^ this is insignificant for the precisions we obtain (>25‰, 1 SD) and we use this reference given its similar matrix properties to the main object of our attention, namely chondritic meteorite matrices. Map data are thus reported as *δ*^*x*/48^Ti_H4-OC_ as defined in eqn (1).1
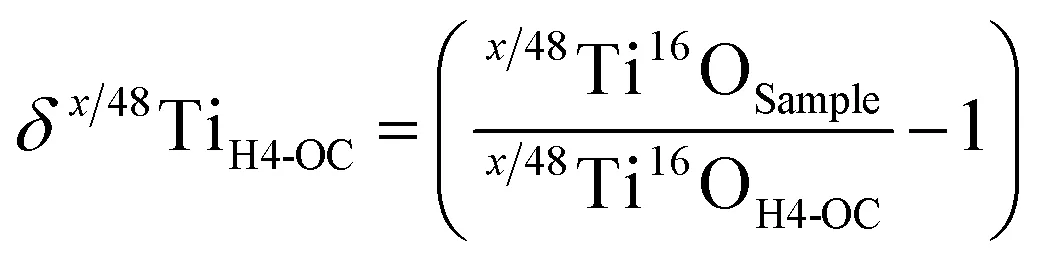
For homogenous standards each analysis is internally normalised to constant ^47^Ti/^48^Ti to correct for instrumental drift. To achieve this normalisation, a fractionation factor, *β*, is calculated using measured ^47^Ti^16^O/^48^Ti^16^O values and a reference value^29^ for the isotope ratio *i.e.*^47^/^48^Ti_Niederer_ (eqn (2)). This implicitly assumes that all fractionation occurs when the ion species is monatomic (^46–50^Ti^+^, ^44^Ca^+^, ^51^V^+^, ^52^Cr^+^) before the reaction with oxygen *i.e.*, in the plasma interface. The fractionation parameter (*β*) for a homogeneous standard is calculated from the mean ratios of the ∼1000 individual integrations:2
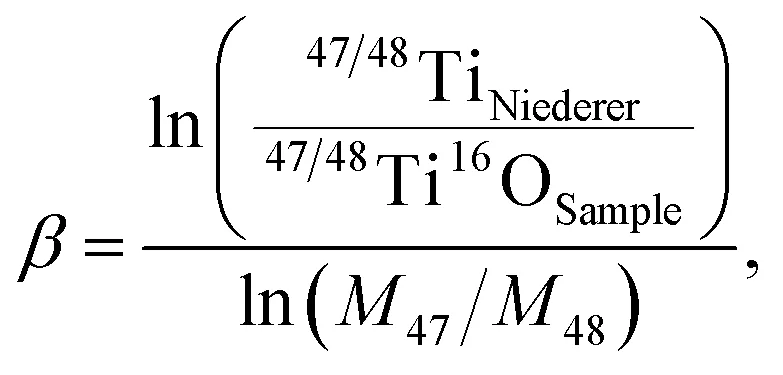
where *M*_47_ and *M*_48_ are the exact masses of ^47^Ti and ^48^Ti. Note that since ^49^Ti is not effectively measured we do not follow recent convention of normalising to ^47^Ti/^49^Ti. Internally normalised, homogeneous standards are then externally normalised to the internally normalised H4-OC to correct for non-exponential components of mass bias and these data are reported:3
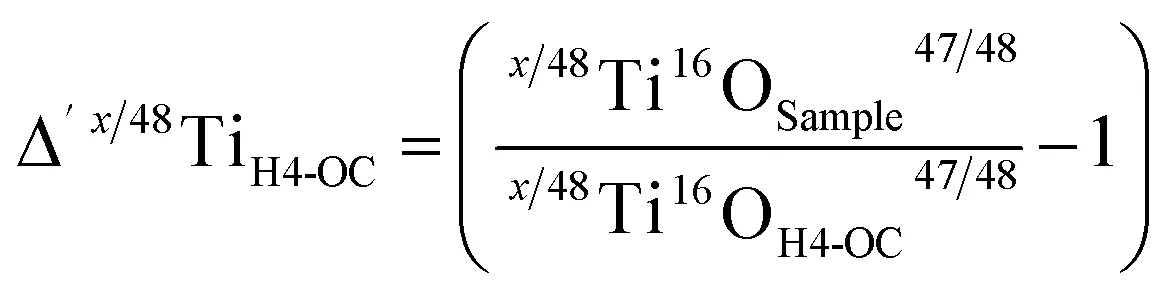


We do not internally normalise sample pixels, given we are looking for presolar grains for which we cannot assume any Ti isotope ratio being constant.

#### 2.6.5 Cr, V corrections

We further use the fractionation factor, *β* (eqn (2)), in the correction of contributions of ^50^Cr^16^O^+^ and ^50^V^16^O^+^ interferences on ^50^TiO^+^ using measured ^52^Cr^16^O^+^ and ^51^V^16^O^+^ intensities. Namely we modify reference values for ^50^Cr/^52^Cr and ^50^V/^51^V to account for instrumental mass bias using the calculated *β*, assuming *β*_Ti_ = *β*_Cr_ = *β*_V_, according to eqn (4):4
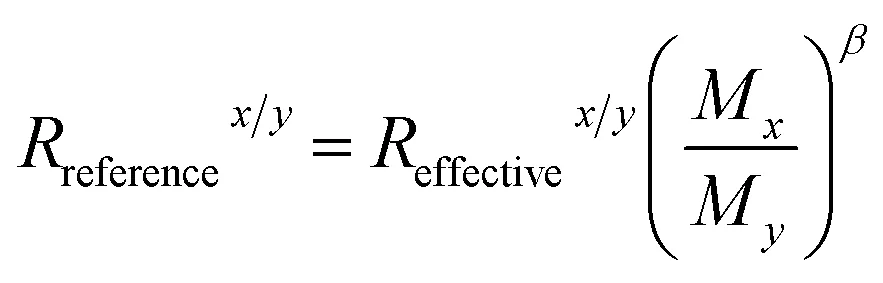
where *x*/*y* are either ^50^V and ^51^V or ^50^Cr and ^52^Cr. This approach makes two implicit assumptions. Firstly, it models elemental rather than oxide fractionation, implying a dominant fractionation in extraction of ions from the plasma, not within the collision cell. Secondly, it assumes a terrestrial Cr and V isotopic composition of the interferences and thus, for sample analyses, their derivation from the surrounding matrix, not presolar grains. This is plausible given the typical size of *in situ* detected presolar grains (∼200 nm) is much smaller than the diameter of our ablation pits (2–3 μm). Moreover, the CrO and VO corrections are often minimal, due to their limited formation in the Proteus collision cell. For each pixel on meteorite maps the Cr and V contributions are corrected using the *β* obtained from the means of bracketing H4-OC.

### 2.7 Dead time effects

Ion counting is subject to artifacts such as the counting system dead time^30^ and non-linear effects. For a time-interval, *τ*, after an ion is detected, the counting system is “dead”, that is, any ions arriving during this interval (the dead time) cannot not be counted (∼70 ns for the CDDs and ∼23 ns for the central SEM). The consequent loss of ions to the counting system is called the dead time effect, which is, to first order, corrected for by the software. This correction is a function of the expected ion arrival rate, estimated by dividing the measured counts by the integration time, *i.e.* the observed mean arrival rate. Clearly, this observed rate is only an approximation to the expected rate and, especially in cases where the ion source intensity changes on timescales comparable to, or shorter than, the integration time, using the observed rate as a basis for this correction results in an inaccurate dead time correction.

The more disparate the abundances of a pair of isotopes, the more greatly their measured ratio is affected by dead time, due to their contrasting intensities or numbers of ions counted. Thus, ratios of isotopes with greater abundance differences are more sensitive to dead time correction inaccuracies, compared to ratios of isotopes with more equal abundances, assuming similar dead times for the corresponding detectors. For example, ^48^Ti is ∼16 times more abundant on the Earth than ^50^Ti, hence, the ^48^Ti^+^ signal will have a (relative) dead time correction ∼16 times greater than that of the ^50^Ti^+^.

**Fig. 4:**
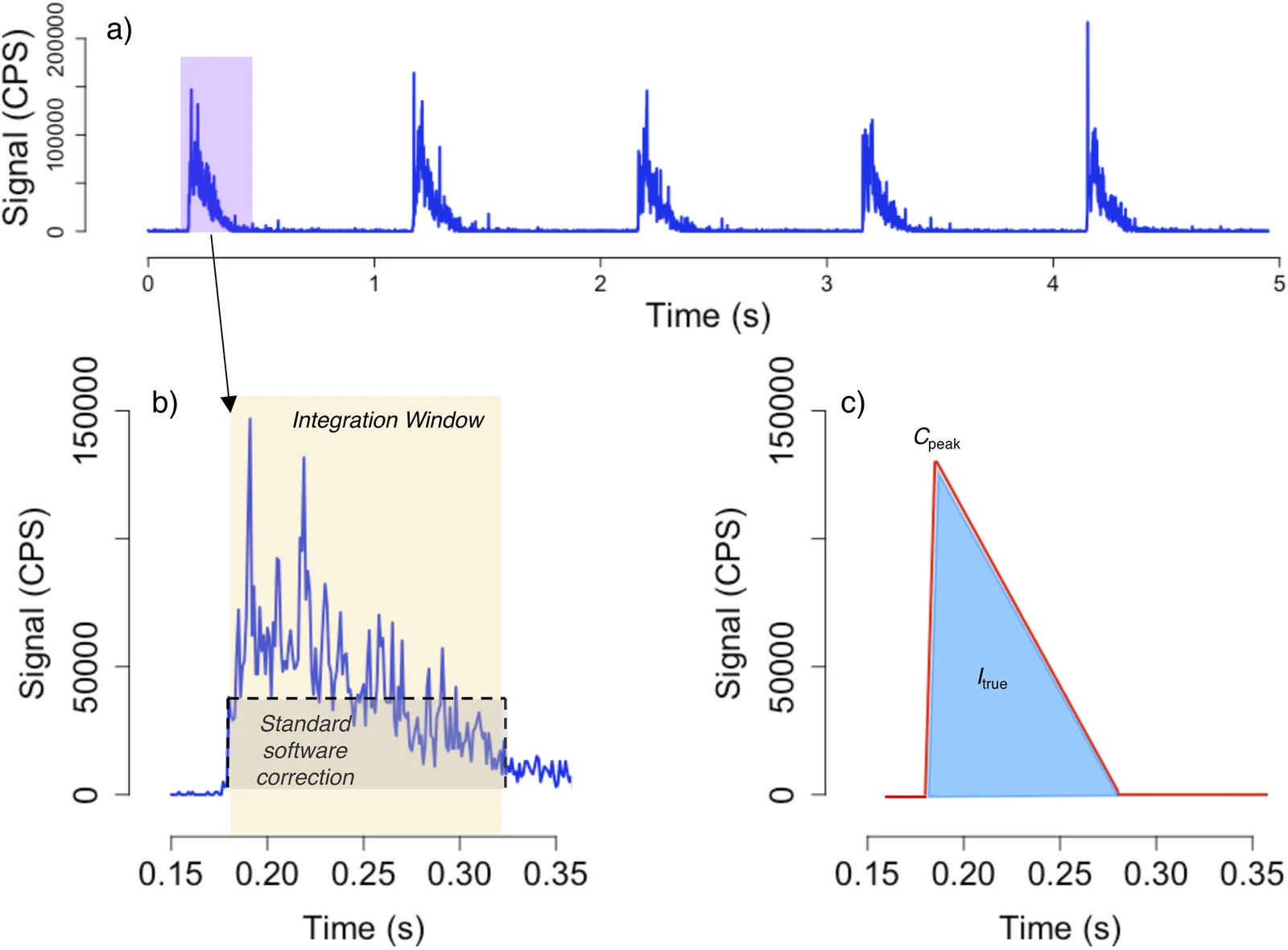
Characteristic signal pulses measured on Proteus (a and b) are the measured pulses on ion-counters using an ARIS sample transfer with laser ablation; and (c) is the idealised pulse shape used in the deadtime model in text, that gauges the non-linearity caused by changes in ion arrival times over the measured pulse.

One laser shot liberates analyte aerosols which are delivered to the plasma *via* the ARIS delivery system. The aerosols arrive at the plasma with a temporal spread of ∼0.1 s, with the majority arriving within 0.05 s, as shown in Fig. 4. The inaccuracy of the software dead time correction is investigated by modelling the ion signal pulse as an asymmetric triangle of width Δ*t* = 0.1 s and peak arrival rate of *C*_peak_. The total number of ion arrivals is,5
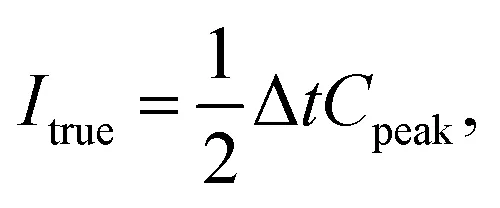


*i.e.*, the area under the triangular signal pulse.

Firstly, we shall consider only the rising part of the signal pulse. If the rise-time is Δ*t*_1_, the count rate as a function of time, *C*(*t*), is given by,

6 *C*(*t*) = *C*_peak_*t*/Δ*t*_1_; 0 < *t* ≤ Δ*t*_1_.

The measured rate, accounting for dead time losses, is given by,

7 *C*_measured_(*t*) = *C*/(1 + *τC*)

8 *C*_measured_(*t*) = (*C*_peak_*t*/Δ*t*_1_)/(1 + *τC*_peak_*t*/Δ*t*_1_); 0 < *t* ≤ Δ*t*_1_.

where we have substituted for *C via*eqn (6). The integral of the above function from *t* = 0 to *t* is,9
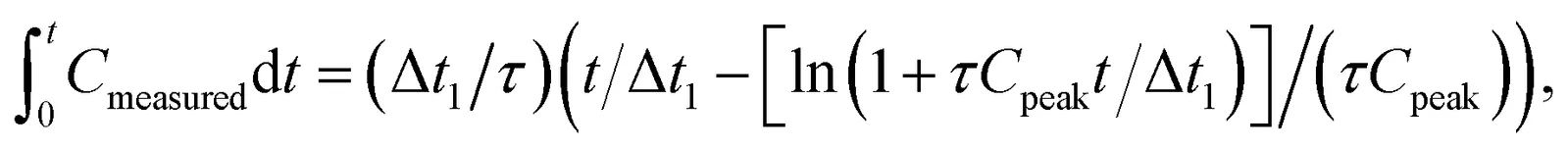
which can be confirmed by recovering eqn (8) by differentiation. Evaluating the integral at *t* = Δ*t*_1_ gives the number of detected ions during the rising part of the signal,

10 *I*_1_ = (Δ*t*_1_/*τ*)(1 − [ln(1 + *τC*_peak_)]/(*τC*_peak_)).

The falling part of the signal can be treated similarly, hence, summing the two, the total number of ions counted is given by,

11 *I*_measured_ = (Δ*t*/*τ*)(1 − [ln(1 + *τC*_peak_)]/(*τC*_peak_)).

Note that, in the limit *τ* → 0, we must have *I*_measured_ → *I*_true_ (eqn (1)). Substituting for the logarithm in eqn (11) the leading two terms of its Maclaurin series readily yields this result.

With the software dead time correction turned on, the reported count rate is given by,

12 *C*_reported_ = *C*_obs_/(1 − *τC*_obs_),

where *C*_obs_ is the mean observed count rate over the integration time, *t*_int_. Assuming an integration time longer than the pulse duration and, furthermore, that just one pulse lies entirely within one integration, we have,

13 *C*_obs_ = *I*_measured_/*t*_int_.

Eqn (11)–(13) comprise our simple model of the reported count rate, *C*_reported_, as a function of peak pulse count rate, *C*_peak_. For peak pulse count rates for ^48^Ti^+^ from 1 to 5 × 10^6^, and corresponding peak pulse count rates for the other isotope's ions based on ref. 29 isotopic abundances, we modelled the measured isotope ratios and calculate the relative deviations from the reference ratios. Other parameters in the model are *t*_int_ = 0.131 s, Δ*t* = 0.1 s and dead times of 23 ns for the ^48^Ti^+^ detector and 70 ns for the detectors of all other Ti isotopes.

**Fig. 5:**
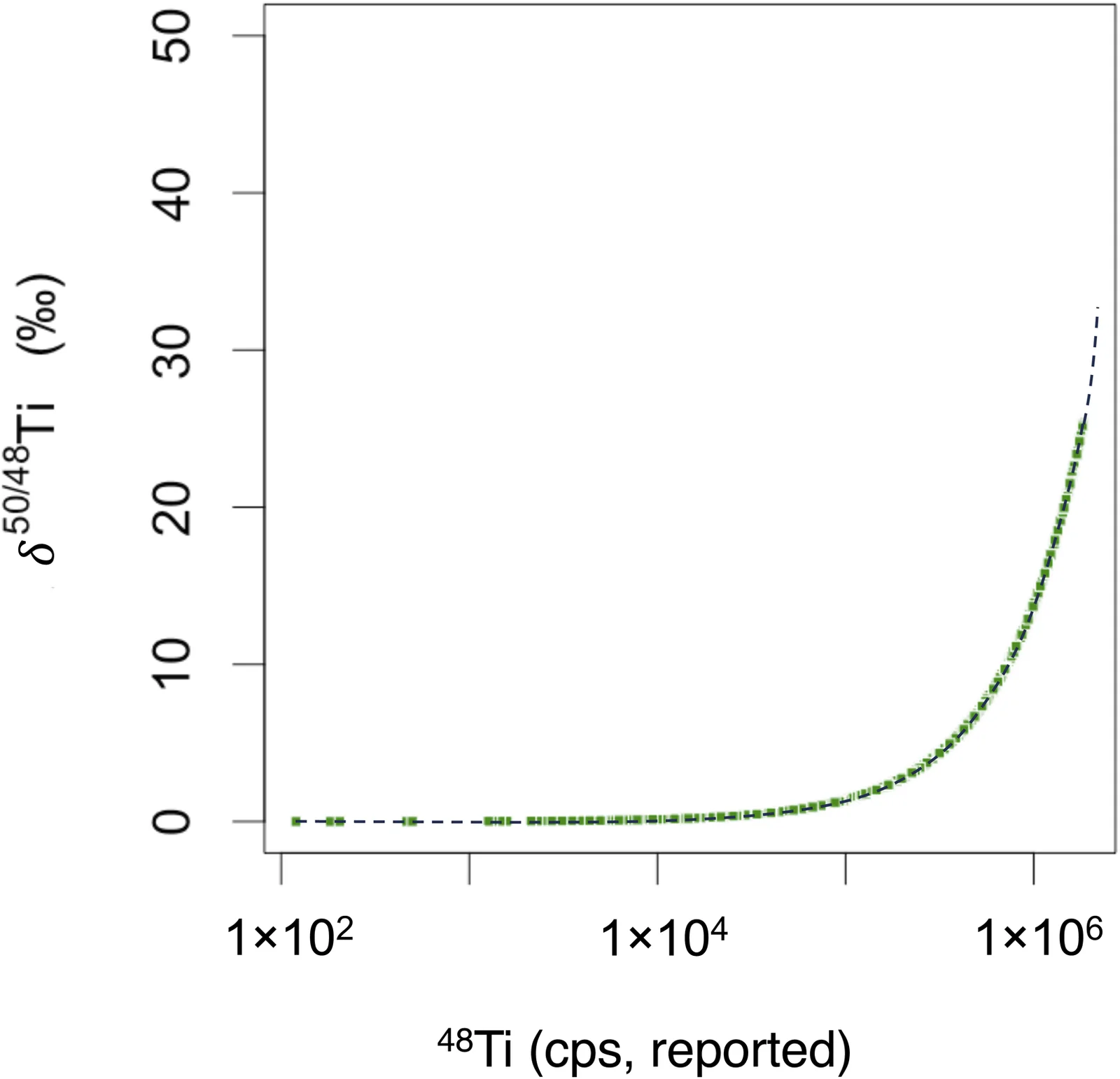
Effect of inaccuracy of dead time correction on ^50^Ti/^48^Ti ratio (expressed in parts per thousand difference from true) at different measured intensities as predicted by our model. ^50^Ti is measured on a detector with a deadtime of 70 ns, while ^48^Ti is measured on a detector with a deadtime of 23 ns.

Our model shows that the inaccuracy due to limitations of the dead time correction is small (<5‰) for most of the sample data, which have counts rates below 100 000 cps of ^48^TiO (Fig. 5). Moreover, associated inaccuracy in the ratios can be cancelled out by bracketing to the standard, but this is only the case if the intensities of the sample and bracketing standards are the same. While the majority of the sample data have intensities akin to the bracketing standard mean intensity ∼75 000 cps ^48^TiO^+^, sample count rates of up to 500 000 cps of ^48^TiO^+^ require a correction of up to 15‰ to account for the inaccuracy induced by dead time (Fig. 5). We therefore implement a correction for dead time inaccuracy according to our model for sample data points with ^48^TiO^+^ intensities above 200 000 cps.

## 3 Results and discussion

### 3.1 Elemental interference reduction

Reduction in the monitored isobaric interferences from Ca, Cr, and V on Ti isotopes is crucial to obtain accurate *in situ* presolar Ti measurements. The more efficient production of TiO^+^ relative to CaO^+^, VO^+^ and CrO^+^ under the tuned conditions of our collision cell results in marked reductions (up to 99%, 70% and 99%, respectively) of the magnitude of interfering ^48^Ca^16^O^+^, ^46^Ca^16^O^+^, ^50^V^16^O^+^ and ^50^Cr^16^O^+^ on isobaric TiO^+^ species relative to measurements as monoatomic species. The most critical effect is for ^50^Cr, where a solar ^50^Cr/^50^Ti ratio is reduced from ∼5 to 0.05 (see Table 2). However, the interference reduction can be tuning dependent and thus varies slightly between sessions. It is found that detuning the TiO^+^ signal from optimal intensity is required to achieve maximum interference reduction. The torch position is particularly important in reducing the Cr^+^/Ti^+^, with peak Ti and peak Cr ionisation regions in the plasma differing considerably. Addition of nitrogen add gas to the plasma complicates the scenario. While increasing overall sensitivity, the peak ionisation positions in the plasma begin to overlap. A more negative plasma extraction voltage increases Cr^+^ compared to Ti^+^. Within the collision cell, interference reduction largely is achieved by tuning the O_2_ and He gases. Too much O_2_ increases CrO^+^ and VO^+^ markedly and thus this is avoided.

**Table 2:** Isobaric interference reduction factors using Proteus

Element	Ca/Ti	^44^Ca/^48^Ti	^46^Ca/^46^Ti	^48^Ca/^48^Ti	V/Ti	^51^V/^50^Ti	^50^V/^50^Ti	Cr/Ti	^52^Cr/^50^Ti	^50^Cr/^50^Ti
CI chondrite	21.90	0.62	0.011	0.056	0.13	2.52	0.0063	6.31	102.10	5.29
CI chondrite after collision cell	0.22	0.0062	0.00011	0.00056	0.039	0.76	0.0019	0.063	1.02	0.053
Percentage reduction	99%				70%			99%		

#### 3.1.1 Non-presolar interference effects

**Fig. 6:**
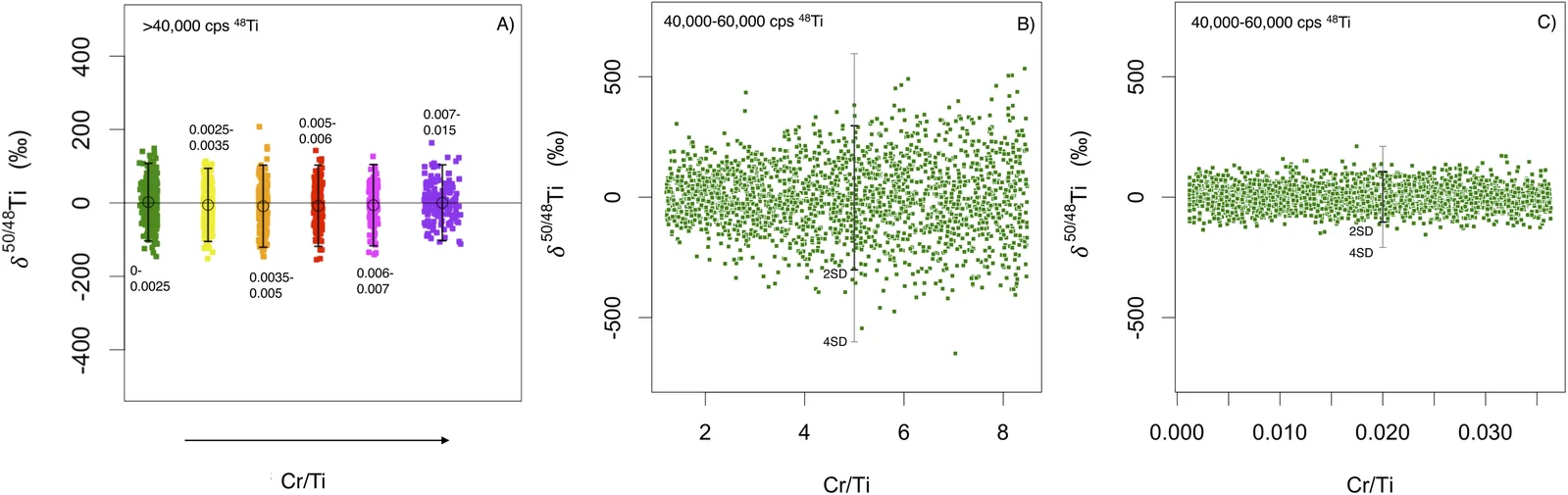
(A) Repeats of the H4 ordinary chondrite nanopowder *δ*^50/48^Ti where reported intensity is greater than 40 000 cps on ^48^Ti *versus* Cr/Ti ratios used for correction (binned by Cr/Ti ratios; black text), calculated from the interference monitor intensity and nominal ratios adjusted for mass bias as described above. (B and C) Modelled *δ*^50/48^Ti with 40 000–60 000 cps count rate on ^48^Ti and error associated with count rates propagated through the Cr correction based on a Poisson distribution. There is a drastic decrease in the spread of the data when the Cr/Ti correction is reduced from CI like ratios (1–8) in (B) to those typical after Proteus has removed the Cr (0.001–0.03) in (C). Black error bars show 2SD of ratios and grey 4SD. Cr/Ti ratios are converted from ^52^Cr/^50^Ti using nominal isotope abundances.

*In situ* oxygen isotope mapping shows presolar oxide and silicate grains (likely the dominant host of ^50^Ti anomalies) are commonly ∼200 nm in size^31^ and so our ablation of a 2–3 μm laser pit very likely dilutes presolar material with surrounding matrix. Thus, an interference correction is applied to sample pixels assuming the majority of Ca, V and Cr is from this solar-like, non-presolar, matrix (Section 2.6.5). This approach is similar to previous NanoSIMS techniques, but our interference corrections are smaller, and we thus minimise our inaccuracy if the assumption that our interferences are dominantly solar is wrong (see below for further assessment of the influence of anomalous isotopic ratios in interference corrections). We also try to minimise the effect of any remaining interferences by filtering out individual sample points with measured ^44^CaO/^48^TiO, ^50^VO/^50^TiO, and ^50^CrO/^50^TiO greater than 0.01, 0.01, and 0.03 respectively, which results in a limited (<10‰) effect from these interferences on the reported TiO ratios (ESI Fig. 2†). This typically excluded <15% of all data in the sample maps but varies slightly from session to session depending on the exact amount of interference reduction. Minimising the Ca, V and Cr also decreases the apparent noise in the data reducing the spread in the data (Fig. 6).

### 3.2 Accuracy and reproducibility of solar materials with interference reduction

#### 3.2.1 Effectiveness and repeatability of interference reduction on different compositions and matrices

**Fig. 7:**
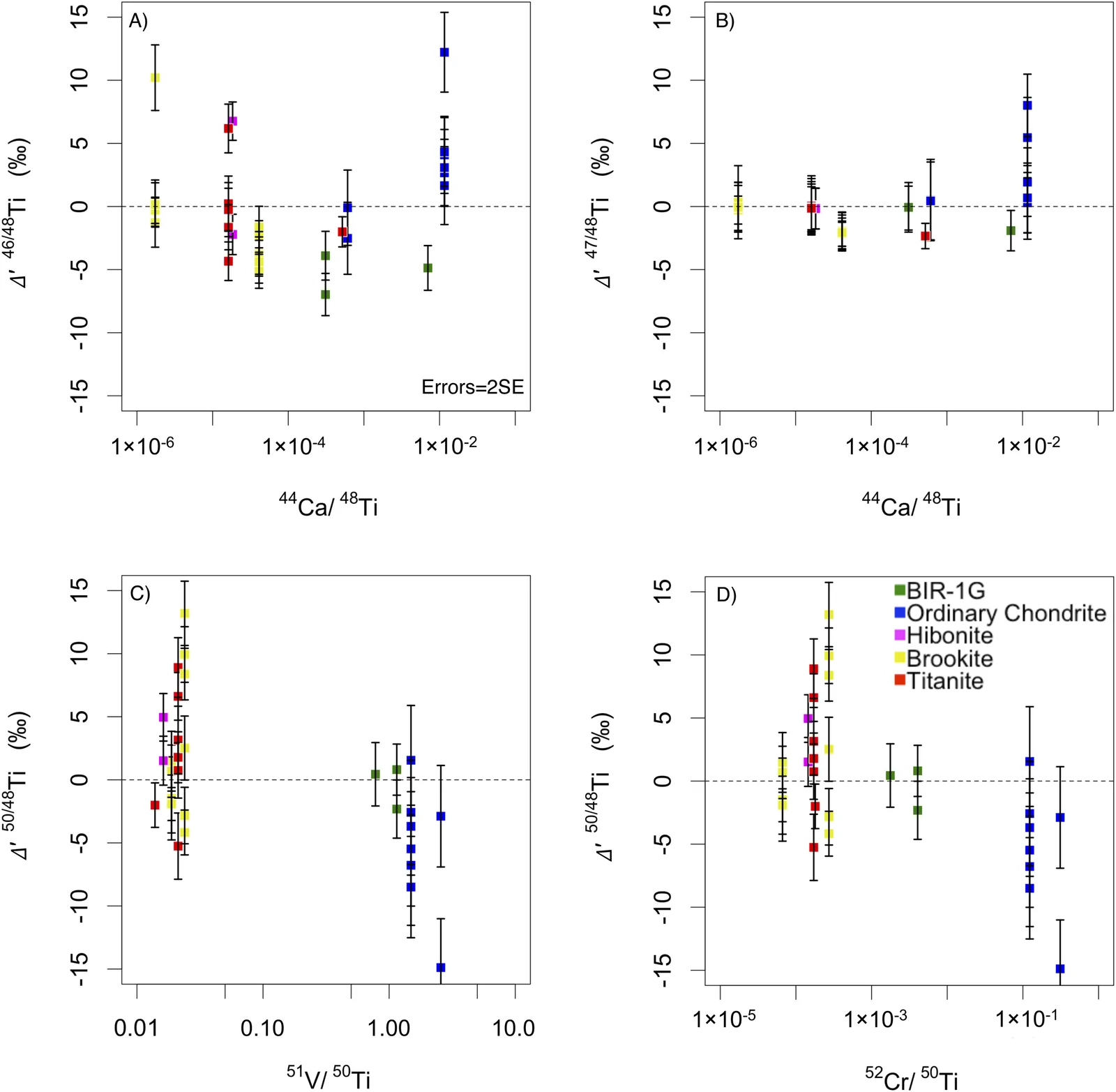
Assessment of accuracy of elemental interference corrections in two different sessions, 18 months apart, using standards with different relative abundances of interfering elements: brookite (yellow), H4 nanopowder (blue), BIR-2G (green), hibonite (pink) and titanite (red). Each datum represents the mean of 1000 single integrations. Error bars are internal 2SE. Normalised to bracketing H4-OC nanopowder. (A and B) *Δ*′^46/48^Ti and *Δ*′^47/48^Ti against measured ^44^Ca/^48^Ti. (C and D) *Δ*′^50/48^Ti against measured ^51^V/^50^Ti and ^52^Cr/^50^Ti respectively.

**Fig. 8:**
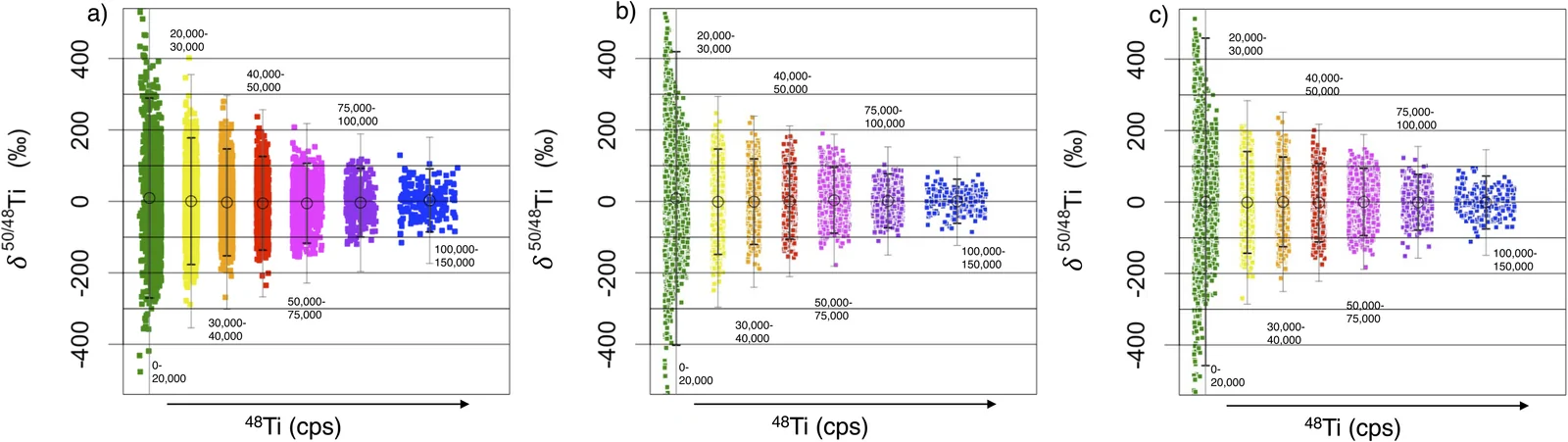
*δ* ^50/48^Ti plotted against ^48^Ti intensity in counts per second (binned into ranges) for (a) repeats of the H4 ordinary chondrite nanopowder and (b and c) for modelled data with pure counting statistical errors based on a Poisson distribution, with and without interference correction, as described in text. Open circles represent mean ratios. Black and grey error bars show 2SD and 4SD of ratios respectively within each bin.

To assess accuracy and reproducibility across varying magnitudes of interference correction and matrix compositions we have plotted internally normalised, mean Ti isotopic compositions of our different standards with their associated 2SE of the 1000 integrations for each mean (Fig. 7). All standards are also externally normalised to bracketing H4-OC nanopowder measurements, which are performed every 5–15 minutes in the analysis routine (Section 2.5). Reported measurements are bracketed to preceding and subsequent H4-OC analyses. These rock standards, homogeneous minerals and the powdered chondrite, all yield *Δ*′^*x*/48^Ti_H4-OC_ that cluster around the expected value of zero mostly within their internal 2SE and all within the long-term reproducibility of ±9‰, ±5‰, and ±12‰ (2SD) for *Δ*′^46/48^Ti^16^O_H4-OC_, *Δ*′^47/48^Ti^16^O_H4-OC_, and *Δ*′^50/48^Ti^16^O_H4-OC_, respectively (Fig. 7). The agreement of different standards with varying chemical and material matrices shows that the technique can be replicated across these different compositions to a level of ∼±10‰. Complex rock compositions, for example BIR-1G, give the same isotopic Ti ratios to essentially pure Ti-minerals, *e.g.* Brookite. Any intrinsic inaccuracies in our approach are therefore small compared to the counting statistical precisions of individual pixels (>±100‰ 2SD, see Fig. 8).

#### 3.2.2 Error estimation on single pixels from standards

The likely error of a single pixel on isotopic maps is hard to estimate given no repeats are possible. The best estimate of uncertainty is given by the internal reproducibility of the individual pixels (2SD) of the H4-OC nanopowder. The H4-OC nanopowder is used because of its similar chemical matrix to target samples which thus incorporate a similar error from the interference and blank corrections.

The *δ*^50/48^Ti of all H4-OC pixels from 10 sessions (10 000 individual integrations) are plotted in groups binned by count rate (Fig. 8). Samples are binned as the signal intensity of the ablated nano-powder is quite variable, following a normal distribution about a mean of ∼75000 cps. We interpret this variability to reflect sample heterogeneity at the 2–3μm length scale of laser ablation, implying that the nano-milling failed to homogenise fully the different components in the H4-OC standard. Given uncertainty in pixel *δ*^50/48^Ti is a strong factor of count-rate, it is useful to compare binned individual analyses of comparable count rate.

We compare our single pixel measurements of H4-OC with a Monte Carlo simulation of counting statistical uncertainty, spanning the measured range in Ti intensities, Cr/Ti and V/Ti. Namely, a ^48^Ti intensity is randomly generated and from that we also produce intensities for ^46^Ti, ^47^Ti, ^48^Ti and ^50^Ti based on reference input ratios.^29^ Using RStudio, these model data are passed through a Poisson distribution, scaled to 
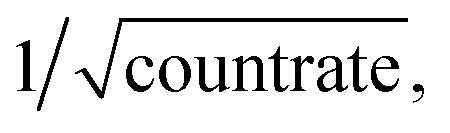
 to produce appropriate random uncertainties that are added or subtracted from the initial intensities. These uncertainty perturbed intensities are used to generate new ratios before normalisation to the original reference input ratios. By repeating this 1000 s of times, we produce a representation of the possible distribution of data given the applicable counting statistics of a real analysis (Fig. 8b). In addition, the effects of V^+^ and Cr^+^ signal subtraction on ^50^Ti^+^, are modelled using ^50^V/^50^Ti and ^50^Cr/^50^Ti that are selected randomly between values of 0.0001 and 0.03. The generated ^50^V^+^ and ^50^Cr^+^ intensities are, as above, passed through a Poisson distribution to give an associated random error associated with their count rates. The ^50^V^+^ and ^50^Cr^+^ signals are then subtracted from the ^50^Ti^+^ intensity. This gives an idea of any increase in the spread of the data caused by these interference corrections (Fig. 8c).

Fig. 8 shows how the reproducibility of the H4-OC nanopowder tablet integrations varies with increasing signal intensity. Fig. 6 shows how the reproducibility of the integrations varies with increasing interference corrections. The modelled data, with a Poisson distribution of variability based solely on count rate, replicates the measured H4-OC nanopowder data well (Fig. 8a and b). Thus, the uncertainty in an individual pixel is mostly dependent on the random error associated with varying count rates. Hence the standard deviation decreases significantly with increasing count rate (Fig. 8). The standard deviation in the real data is (inevitably) greater than the modelled data, but only slightly (20–30‰ more, 2SD). The additional variability could be error propagated by the corrections which themselves have an associated random measurement error, most notably the blank or Cr and V subtractions. Including the Cr and V corrections in the model appears to add some noticeable variability (10–15‰) to the modelled data of *δ*^50/48^Ti (Fig. 8c and 6) which suggests that some of the additional noise in the real data is caused by the correction. The error associated with the Cr correction is, however, much diminished by reducing the Cr/Ti using Proteus (Fig. 6).

To ensure that anomalies in the sample are resolvable from random noise, we assume the data follows a normal distribution and advocate that values greater than 4 times the standard deviation (>4SD, *p* < 0.000063, based on nanopowder reproducibility) are probable presolar anomalies; *i.e.* the probability that an ablation analysis of an isotopic normal spot randomly yields a similar anomaly to a true presolar grain (a false positive) is < 1 in a map of ∼10 000 pixels. A similar criterion is used in other studies of presolar grains.^32^ For pixels in sample maps with a count rate >50 000 cps on ^48^Ti, this generally corresponds to anomalies greater than 150‰ in *δ*^46/48^Ti and *δ*^47/48^Ti, and 200‰ in *δ*^50/48^Ti (4SD). However, for count rates much greater than 50 000 cps on ^48^Ti, smaller anomalies are considered resolvable owing to the decrease in standard deviation as shown in Fig. 8. Using this criterion, we do not find any false anomalies in the repeat analyses of standards, including the H4-OC nanopowder (Fig. 8), suggesting that any anomalies found in primitive meteorite mapping are very likely to be presolar.

The generally lower sensitivity of Proteus due to the low plasma extraction voltage compared to the Neoma CC-MC-ICPMS/MS does impart a limitation on precision given the strong relationship between measurement error and sensitivity (Fig. 8). However, the ability of Proteus to exclude more unwanted ions from the collision cell (see Section 2.3) is significant compensation for this loss in sensitivity. An instrument with the same mass-prefilter capabilities as Proteus but able to decelerate the ion beam from a −2000 eV potential to the much lower energy necessary for the quadrupole filter would overcome this. The higher sensitivity would either allow much better detection limits of less anomalous presolar grains for a 2 μm laser resolution, or, allow detection of much smaller grains at a 1 μm laser resolution through less dilution with solar material. At the writing of this paper, no such technology has been developed.

### 3.3 Presolar grain application

#### 3.3.1 Spatial resolution and dilution

**Fig. 9:**
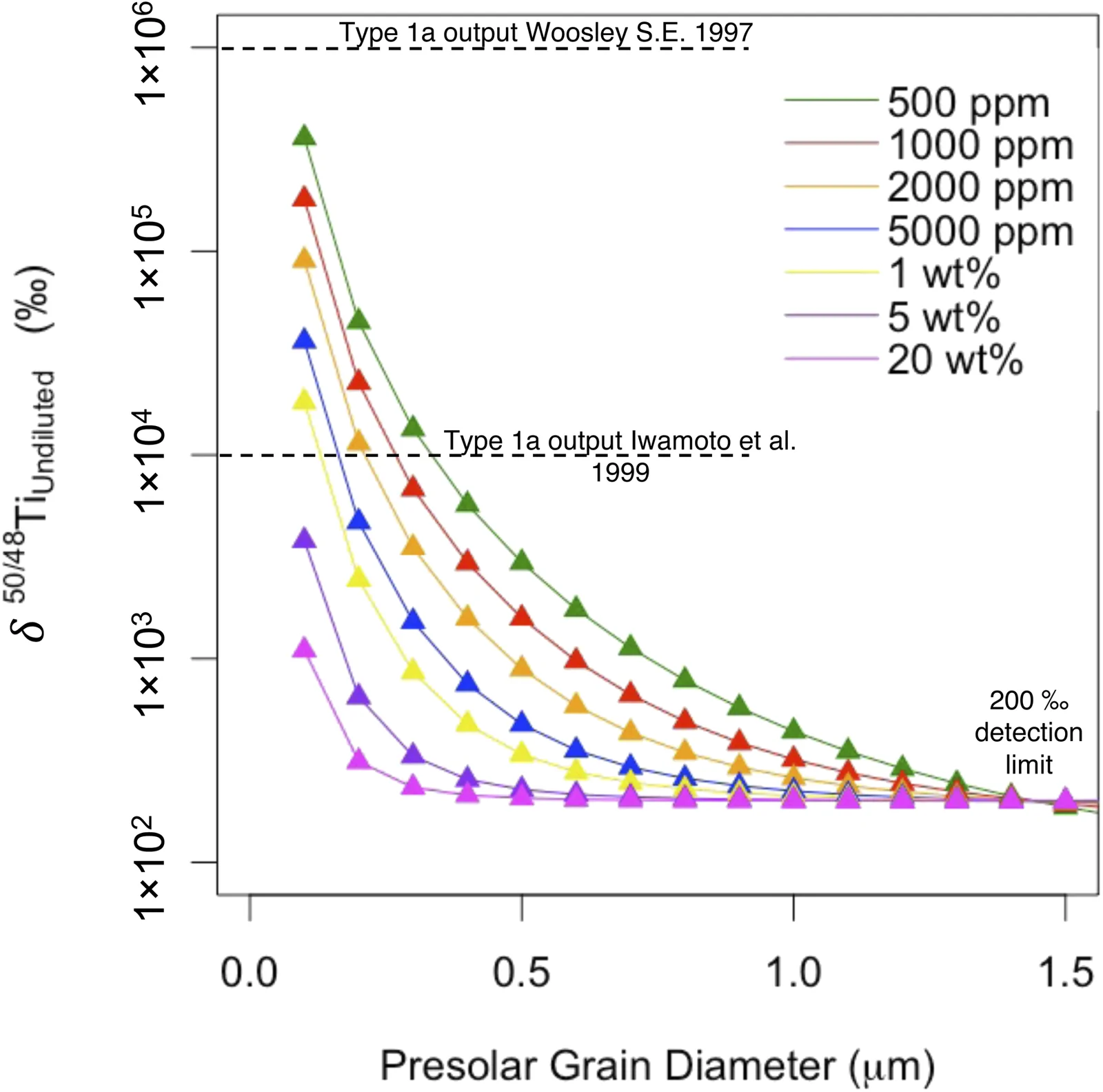
Calculated undiluted presolar *δ*^50/48^Ti for a measured anomaly at our detection limit of 200‰ (we assume a constant volume of ablation for a 2 μm spot of 0.5 μm depth and vary the Ti concentration and diameter of a spherical presolar grain. The matrix Ti concentration that dilutes the presolar grain is taken to be 300 ppm.

As discussed in 3.1.1, using a 2 or 3 μm laser spot, dilutes most ablated presolar grains with solar matrix material during sampling. Thus, all isotopic anomalies measured by our approach must be considered lower limits. The amount of signal dilution depends on the volume of the laser pit, how well the laser pit incorporates the full shape of the grain, and the Ti concentration of the diluting material.^33^Fig. 9 shows how the true *δ*^50/48^Ti of a grain might relate to a measured value of 200‰, close to the limit of detection for a resolvable *δ*^50/48^Ti anomaly (Section 3.2.2). This model assumes a constant matrix composition of 300 ppm^34^ Ti, an ablation volume defined as a 2 μm diameter cylinder with a depth of 0.5 μm, the average depth of ablation and the presolar grain (as a sphere of variable diameter) fully sampled in this volume. Mainstream presolar SiC grains can have up to several 1000 ppm of Ti but only a mean *δ*^50/48^Ti of ∼200‰ and are thus only likely detectable if over 1 μm in diameter^15^ (Fig. 9). This might suggest that they are not as easily detected by our methods as by NanoSIMS and meteorite leaching. The mineralogy of silicate and oxide grains have been discussed previously in the literature, with the majority of oxides identified as spinel or corundum.^35,36^ Spinels and corundum (>50 nm in diameter) with several weight percent of Ti would be detectable (Fig. 9), given previously measured compositions of *δ*^50/48^Ti > 1000‰ relating to possible origins in highly anomalous supernovae.^21,37^ Presolar silicate compositions are still highly debated, but the consensus is that they fall between pyroxene and olivine stoichiometries,^36^ thus they could ideally be detected if such mineralogies contain enough Ti or they also come from highly anomalous supernovae.

#### 3.3.2 Presolar interference effects

**Fig. 10:**
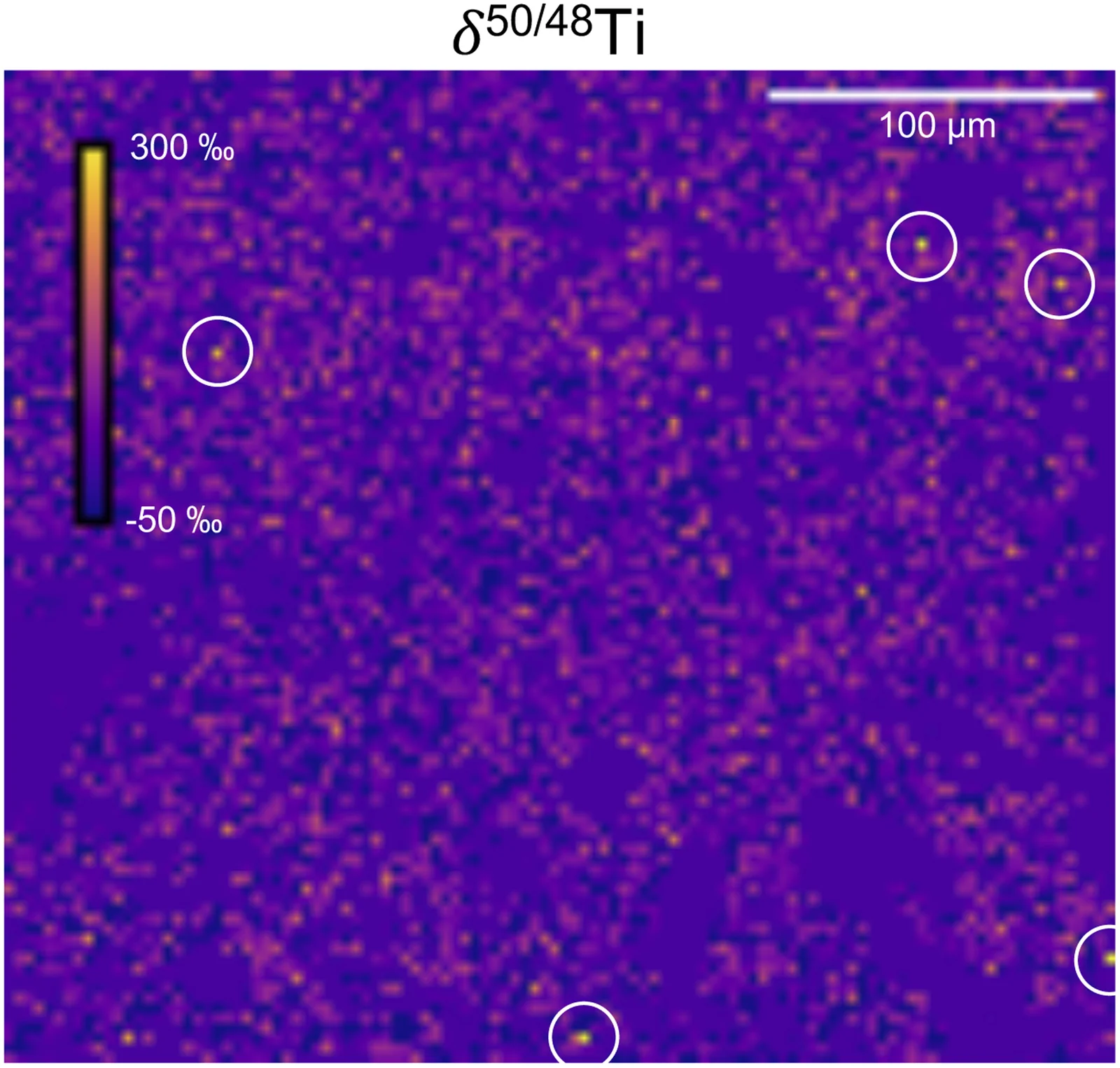
Isotope map of *δ*^50/48^Ti on the C2 ungrouped meteorite Acfer 094 mapped at 2 μm laser resolution exhibiting five presolar anomalies above 250‰, one being >1000‰ (lowest most circle).

**Fig. 11:**
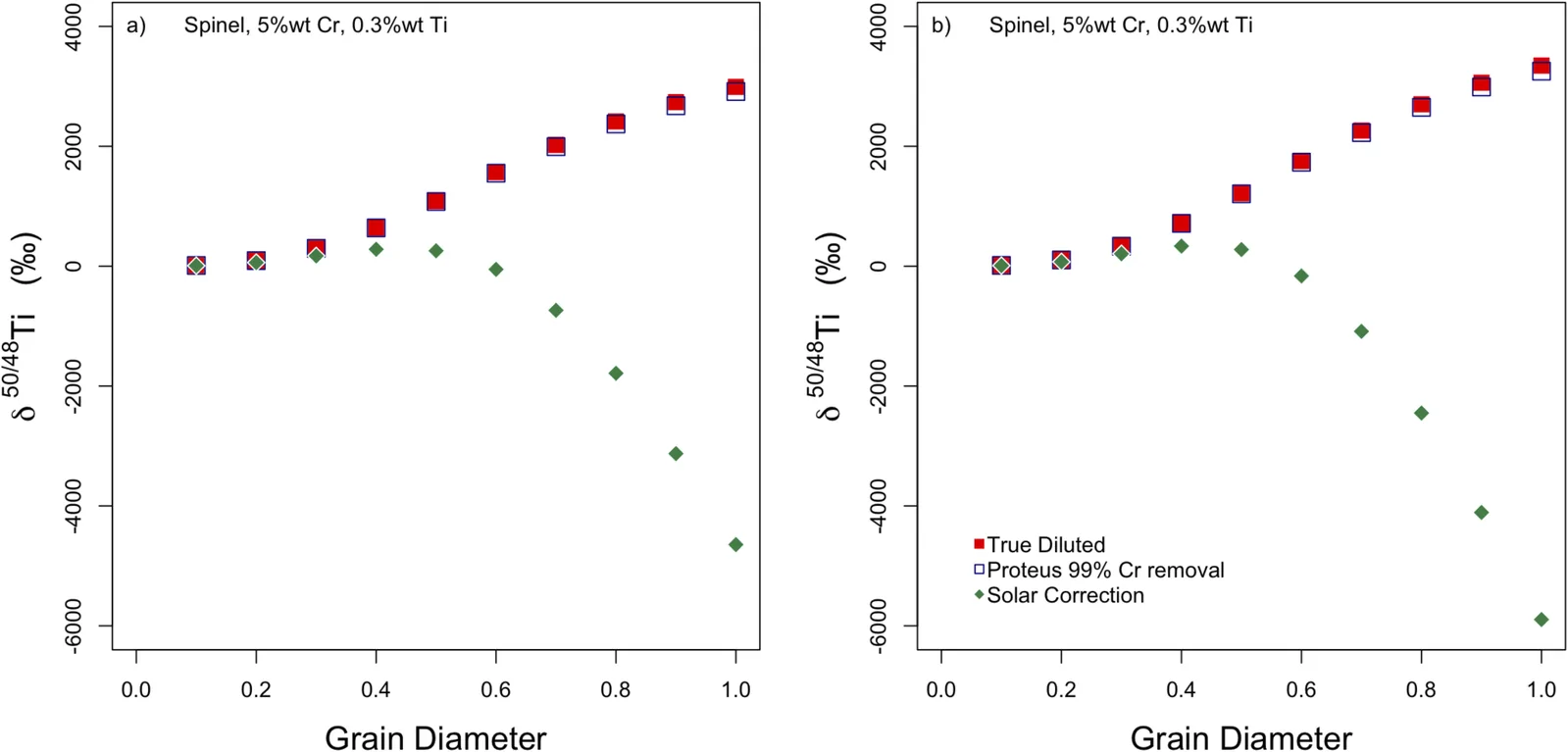
The effect on inferred Ti isotopic composition of a presolar grain analysis from assuming a solar Cr isotopic composition for Cr interference correction. Two grains are modelled with Ti and Cr isotopic compositions from different Type-1a supernovae models (a and b) that are considered a likely source of Solar System Ti anomalies. Bulk compositions of Cr-spinel/oxide grains are assumed. The red squares show the true composition of a presolar grain ablated, diluted with surrounding solar material during ablation of a standard 2 μm pit. Using our technique without Cr removal shows that as the presolar grain size increases, or dilution decreases, the correction using terrestrial ratios becomes more and more inaccurate (green diamonds). Using Proteus to remove 99% of the Cr before a terrestrial correction for Cr (open blue squares) has minimal inaccuracy from the correction. Grain diameter in μm.

It is critical to reduce any possible presolar Ca, V or Cr interferences, as there is no way to determine the unknown presolar isotope abundances of the interfering elements, but we can show the possible effects on reported anomalies. Fig. 11 shows the inaccuracy of interference correction using solar ^50^Cr/^52^Cr for a grain with different presolar isotope ratios from a type 1a supernova. Type 1a supernovae are a very likely environment for the origin of Solar System Ti anomalies^21^ and generally have lower ^50^Cr/^52^Cr than solar. Thus, correcting Cr using solar ratios on a presolar oxide (spinel in this case) with a type 1a signature^4,5^ would likely create inaccurate results (Fig. 10). However, by removing 99% of the Cr with Proteus, any inaccuracy from a terrestrial correction on a presolar ratio becomes minimal. The effect of presolar isotope ratios in the interfering species is something that previous studies did not fully address. While some show that their apparent Ca/Ti, V/Ti, Cr/Ti ratios are low enough that they expect limited effects from other presolar isotopes on their final ratios,^15,21^ they use monitored isotopes to show this, which assumes that the isobaric presolar isotopes correspond with monitor isotopes *i.e.* low ^52^Cr means low ^50^Cr. This assumption may be true, but remains a significant uncertainty. Therefore, direct removal of the element is the most reliable way to observe an un-interfered presolar Ti ratio.

## 4 Conclusions

Within this study we have developed an *in situ* technique to measure Ti isotopic compositions using Proteus, a novel CC-MC-ICPMS/MS. We use a pre-cell mass filter-collision cell capability to react Ti away from its isobaric interferences, as TiO^+^ ions using O_2_ gas, into a mass range cleared by the mass filter. Combining laser ablation with an ARIS and an ion counter array, we can measure Ti in small sample volumes (1–3 μm^3^) as required to detect presolar grains. We measured the Ti isotopic compositions, *in situ*, of different terrestrial samples and one meteorite powder to test the ability of the method to identify presolar signatures from picograms of Ti. Our technique was able to consistently reduce Ca/Ti, V/Ti, and Cr/Ti by approximately >99%, >70% and >99% respectively which allows more accurate measurement of Ti in presolar grains, given uncertainty in the appropriate isotope ratio for interference correction.

This study has shown that we can detect anomalies greater than 150‰ in *δ*^46/48^Ti and *δ*^47/48^Ti, and 200‰ in *δ*^50/48^Ti, with varying amounts of Ca, V, Cr, for count rates of ^48^TiO greater than 50 000 cps (for a 0.131 s measurement). Given the removal of Ca, V, and Cr was so effective, the main limitation on reproducibility and accuracy was the low number of counts associated with the small measurements required for presolar grains.

## Conflicts of interest

There are no conflicts to declare.

## Supplementary Material

JA-040-D5JA00068H-s001

JA-040-D5JA00068H-s002

JA-040-D5JA00068H-s003

## Data Availability

The data supporting this article have been included as part of the ESI.†
