# Correction: *In situ* analysis of titanium isotope ratios in stardust using LA-CC-MC-ICPMS/MS

**DOI:** 10.1039/d6ja90052f

**Published:** 2026-11-06

**Authors:** Kathryn M. M. Shaw, Markus Pfeifer, Benjamin L. L. Coath, Jamie Lewis, Dan Bevan, Christopher D. Coath, Tim Elliott

**Affiliations:** a School of Earth Science, University of Bristol Queens Road Bristol BS8 1RJ UK Kathryn.shaw@bristol.ac.uk; b Thermo-Fisher Scientific (Bremen) GmbH Hanna-Kunath St. 11 28199 Bremen Germany; c Centre for Exploration Targeting, School of Earth Sciences, University of Western Australia Perth Western Australia Australia

## Abstract

Correction for ‘*In situ* analysis of titanium isotope ratios in stardust using LA-CC-MC-ICPMS/MS’ by Kathryn M. M. Shaw *et al.*, *J. Anal. At. Spectrom.*, 2025, **40**, 1566–1579, https://doi.org/10.1039/D5JA00068H.

The authors regret the omission of a grant number in the acknowledgements section of the article.

The full and correct acknowledgements are as below.

This work was supported by a UKRI STFC Studentship (ST/S505262/1) and an ERC grant (ISONEB ERC Adv 321209) at the University of Bristol. The authors would like to thank Prof. Mahesh Anand at the Open University for providing the H4 ordinary chondrite used in this work and Dr Stuart Kearns and Dr Benjamin Buse for their advice and training in the University of Bristol microbeam laboratory.

The Royal Society of Chemistry apologises for these errors and any consequent inconvenience to authors and readers.

