# Supplementary material for: In situ analysis of titanium isotope ratios in stardust using LA-CC-MC-ICPMS/MS
Source: J Anal At Spectrom. 2025 May 15;40(6):1566–79. doi: 10.1039/d5ja00068h (PMC12097295; doi:10.1039/d5ja00068h)
Supplement: JA-040-D5JA00068H-s001 [file JA-040-D5JA00068H-s001.pdf]

Supplementary Information (SI) for Journal of Analytical Atomic Spectrometry.
This journal is © The Royal Society of Chemistry 2025

Supplementary Information

In situ Analysis of Titanium Isotope Ratios in Stardust using LA-CC-MC-ICPMS/MS

Kathryn M. M. Shaw[a,b], Markus Pfeifer[a,c], Benjamin L. L. Coath[a], Jamie Lewis[a], Dan Bevan[a,d], Christopher D. Coath[a], Tim Elliott[a]

[a]School of Earth Science, University of Bristol, Queens Road, Bristol, BS8 1RJ, UK

[b]Department of Earth Sciences, University of Cambridge, Downing Street, Cambridge, CB2 3EQ, UK

[c]Thermo-Fisher Scientific (Bremen) GmbH, Hanna-Kunath St. 11, 28199, Bremen, Germany

[d]Centre for Exploration Targeting, School of Earth Sciences, University of Western Australia, Perth, Western Australia, Australia

*Correspondence Kathryn.shaw@bristol.ac.uk

Oxygen isotope quantification of the gas.

Due to the processes of purifying the oxygen gas used in an analysis for Ti with Proteus, the gas is likely largely mass-dependently fractionated, possibly non-exponentially so. To correct for the minor isotopes on successive TiO masses, the $^{17}O/^{16}O$ and $^{18}O/^{16}O$ ratios of the reaction gas must be known and quantified. Proteus permitted this using the single-mass transmission mode through the pre-filter quadrupole. A single mass of Ti was let through into the collision cell where it formed all three possible types of oxide adduct: $^{x}Ti^{16,17,18}O$. The adducts were exclusively measured on different detectors i.e. $^{46}Ti^{16}O$, $^{46}Ti^{17}O$, $^{46}Ti^{18}O$ were measured on subsequent faraday detectors set up for $^{46}Ti^{16}O$, $^{47}Ti^{16}O$ and $^{48}Ti^{16}O$ without the possibility of any other Ti isotopes interfering. The $^{x}Ti^{16}O$, $^{x}Ti^{17}O$ and $^{x}Ti^{18}O$ signals were then ratioed to each other which provides an estimate of $^{17}O/^{16}O$ and $^{18}O/^{16}O$. This was done for each isotope to look for mass independent behaviour of the Ti-oxygen reaction.

Supplementary Table 1

Sample	Ti (µg/g)	Ca (µg/g)	V (µg/g)	Cr (µg/g)
**TiO_2 (Brookite)**	600000	<100	<100	<100
**Titanite**	240000	200000	<100	<100
**BIR-1G**	5400	95000	300	400
**H4 Chondrite (M12)**	600	12500	100	3600

Supplementary table 1: Standard and reference material approximate concentrations of Ti, Ca, V, and Cr. BIR-1G data compiled from GeoRem database[2] and chondrite data from Wasson and Kallemeyn (1988).

Supplementary Table 2

Introduction System		Min	Max	
**Aridus**	Sweep Ar	2.3	4.2	L/min
	Nitrogen	0.04	1.0	L/min
	Auxiliary Gas	0.75	0.85	L/min
	Nebuliser Gas	0.9	1.0	L/min
**Laser**	He1	0.45		L/min
	He2	0.35		L/min
	auxiliary Gas	0.75	0.85	L/min
	Nebuliser Gas	0.9	1.0	L/min
	Secondary Nitrogen	1.0	4.0	mL/min
**Torch**	Sampling Depth	1.7	4.9	mm
	Horizontal	-1.5	-0.5	mm
	Vertical	-2.1	-0.8	mm
Front End Lenses				
	Extraction	-400	-300	V
	Deflection Entry	-35	-25	V
	Angular Deflection	-450	-380	V
Quadrupole Mass Window				

	RF DAC Offset	-525		
	DC DAC Offset	-520	-425	
	Quadrupole Entry Lens	-170	-120	V
	Quadrupole Focus Lens	-14	3	V
	Quadrupole Exit Lens	-65	-45	V
	Pole Bias	-1		V
Collision Cell				
	Oxygen	2.0	3.0	mL/min
	Helium	3.0	4.0	mL/min
	Drag Entry Top Lens	-25	-23	V
	Drag Entry Bottom Lens	-25	-21	V
	Drag Voltage Lens	-60	-20	V
	CCT Bias	-2		V
	CCT Exit Lens	-10	-5	V
Neptune Lenses				
	Focus	-690	-670	V
	X-Deflection	-4.9	-4.2	V
	Y-Deflection	-3	2	V
	Shape	195	199	V
	Source Offset	-6		V
	Focus Quad	-20		V
Laser				
	Spot Size	1	10	µm
	Fluence	2	7	J/cm^2
	Rep Rate	4	8	Hz
	Scan Rate	10	22	µm/s

Supplementary table 2: Common instrument parameters for Proteus.

Supplementary Figure 1

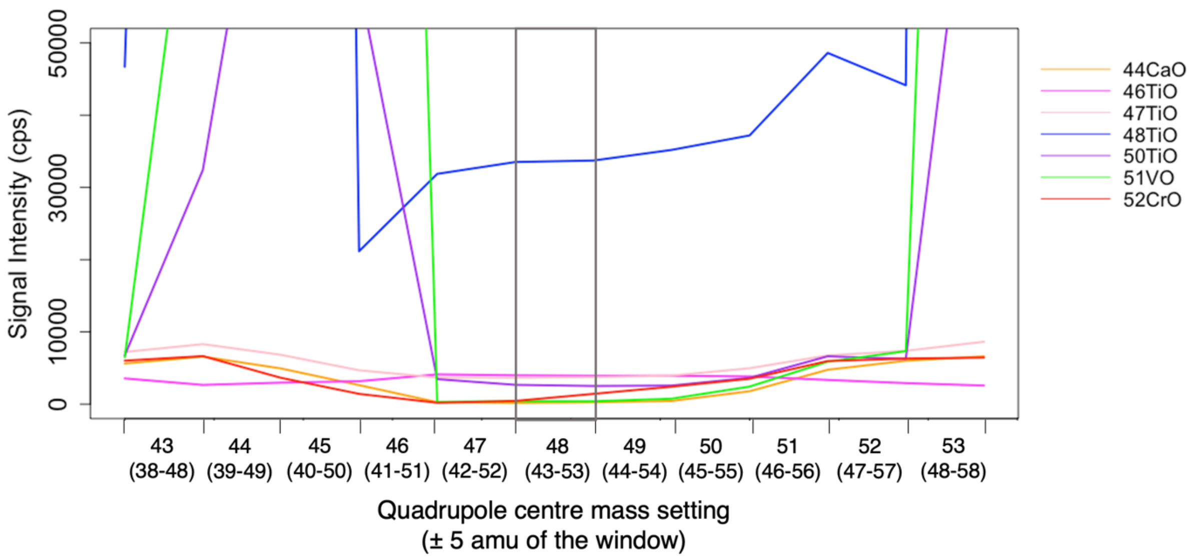

Supplementary Figure 1: Signal intensities at different masses (corresponding to target species for analysis) collected on the multiple detectors during scan across the quadrupole of 10 amu around *u/q* 48 from 43 to 53. Owing to the 10 *u/q* width of the quadrupole mass filter around the central mass, each central mass represents a range of masses allowed to proceed through the mass-prefilter as shown in brackets. For example, this study measures with the quadrupole *u/q* set at 48 therefore allows roughly *u/q* range of 43 to 53 at this setting (black box). From this it is clear that when $^{40}Ar^{+}$ and $^{40}Ar^{16}O^{+}$ are allowed to enter into the collision cell at *u/q* settings 45 and 51 respectively large interference signals are produced, especially on the $^{48}Ti^{16}O^{+}$, $^{50}Ti^{16}O^{+}$, and $^{51}V^{16}O^{+}$ ion counter signals.

Supplementary Figure 2

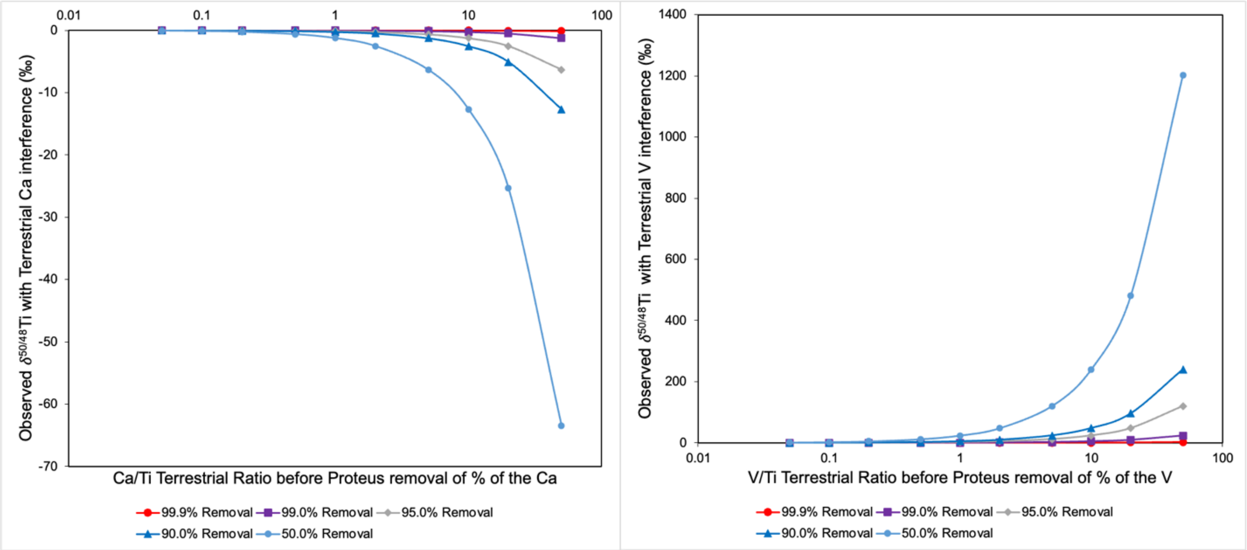

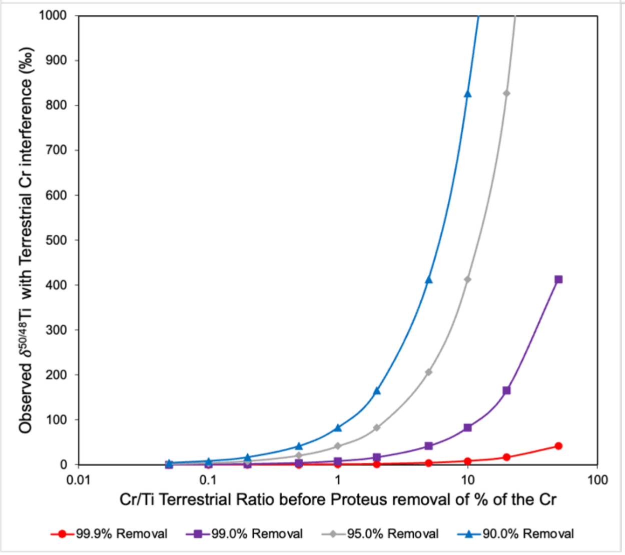

Supplementary figure 2: Model demonstrating the effect of increasing terrestrial Ca, V, and Cr relative to Ti and the apparent $\delta^{50/48}$Ti anomaly created by their isobaric interferences. The model demonstrates how reducing interference/Ti ratios creates more accurate $\delta^{50/48}$Ti, without isobaric corrections. Proteus removes >99.9% of Ca, >97% Cr, and ~70% V.

Supplementary Figure 3

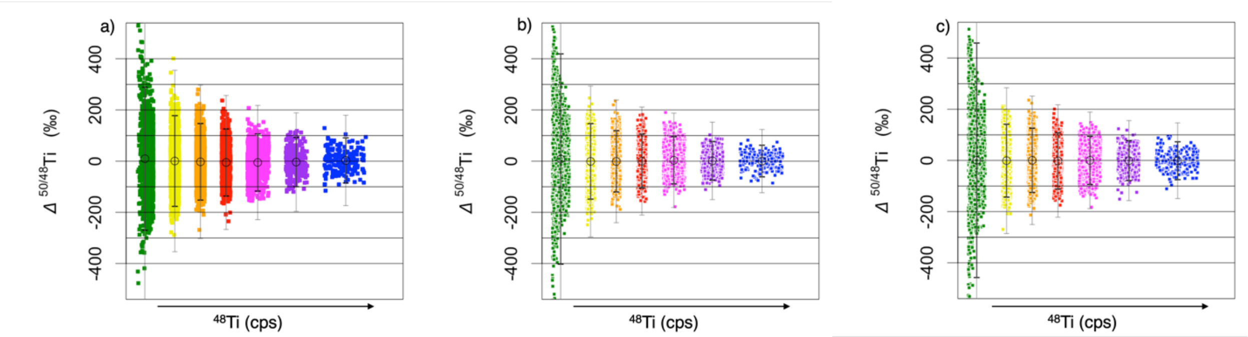

Supplementary figure 3: Δ $^{50/48}Ti$ vs binned reported ^{48}Ti intensity (cps); a) repeats of the M12 ordinary chondrite nanopowder; b) modelled data with random error distribute by a Poisson distribution applied on the intensities of Ti with a deadtime induced inaccuracy as described in text; c) modelled as in b with random V and Cr intensities subtracted from the ^{50}Ti in the range of 0.001—0.03 of the ^{50}Ti intensity. Circle outlines represent mean ratios. Black and grey error bars show 2SD and 4SD of ratios respectively within each bin.

Supplementary Figure 4

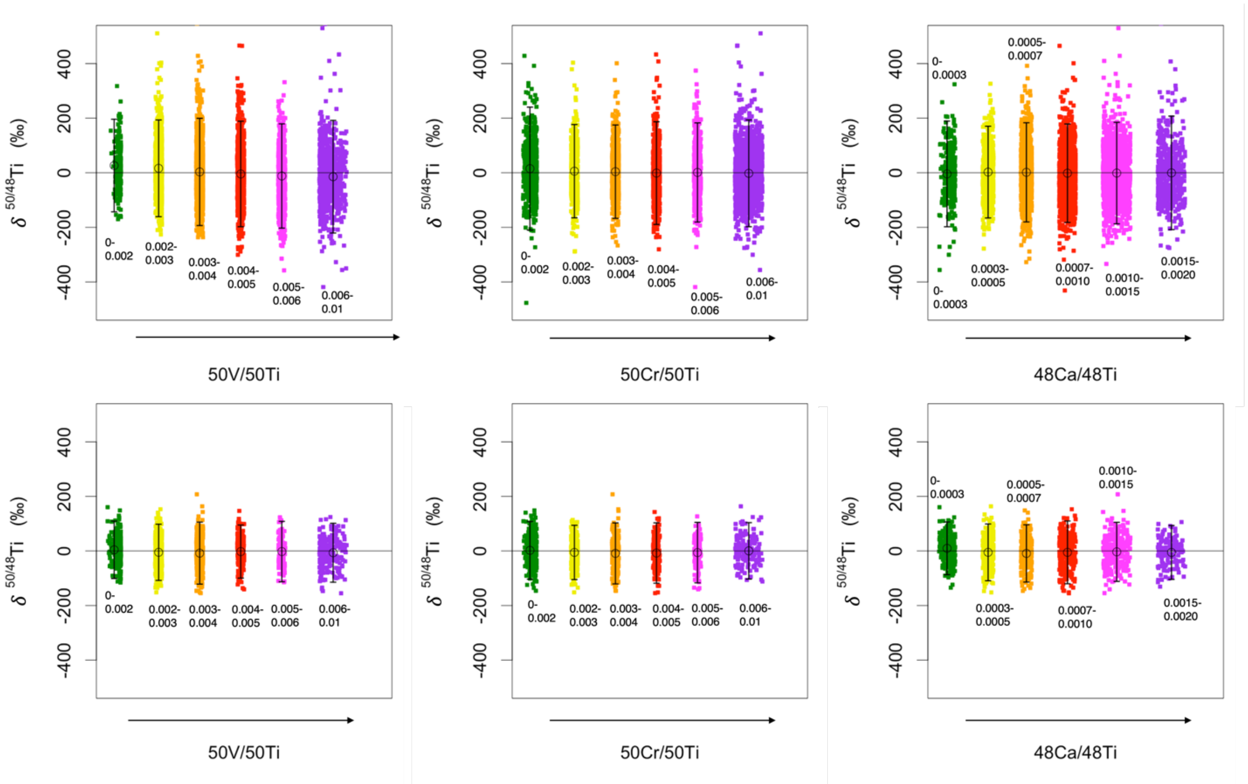

Supplementary Figure 4: $\delta^{50/48}Ti$ compared to monitored interference ratios used for correction (binned ratios; black text), calculated from the interference monitor intensity and nominal ratios adjusted for mass bias; a-c) repeats of the H4 ordinary chondrite nanopowder where reported intensity is less than 50,000 cps on ^{48}Ti; d-f) repeats of the H4 ordinary chondrite nanopowder where reported intensity is greater than 50,000 cps on ^{48}Ti. Circle outlines represent mean ratios. Black error bars show 2SD of ratios within each bin.

Supplementary Figure 5

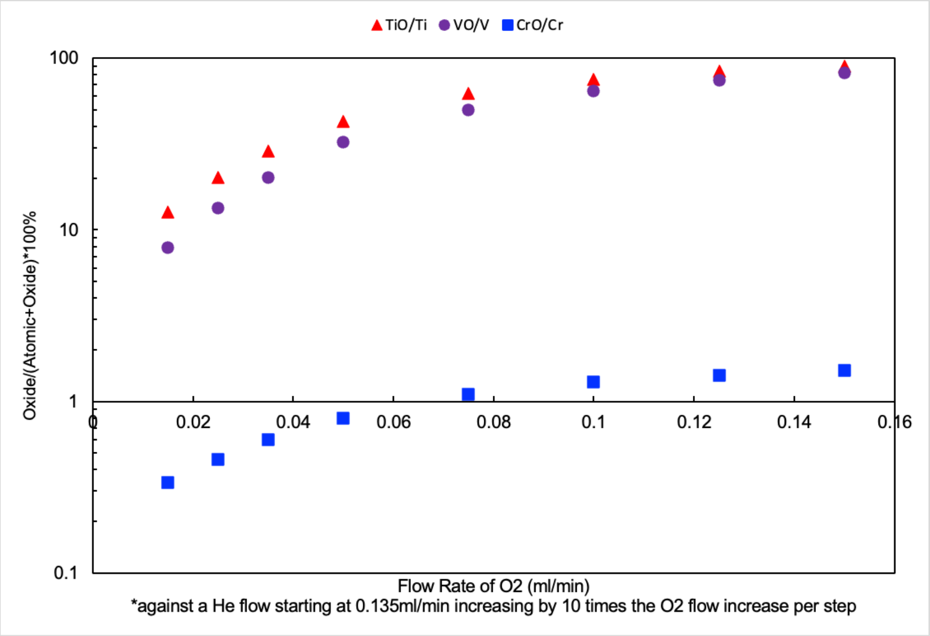

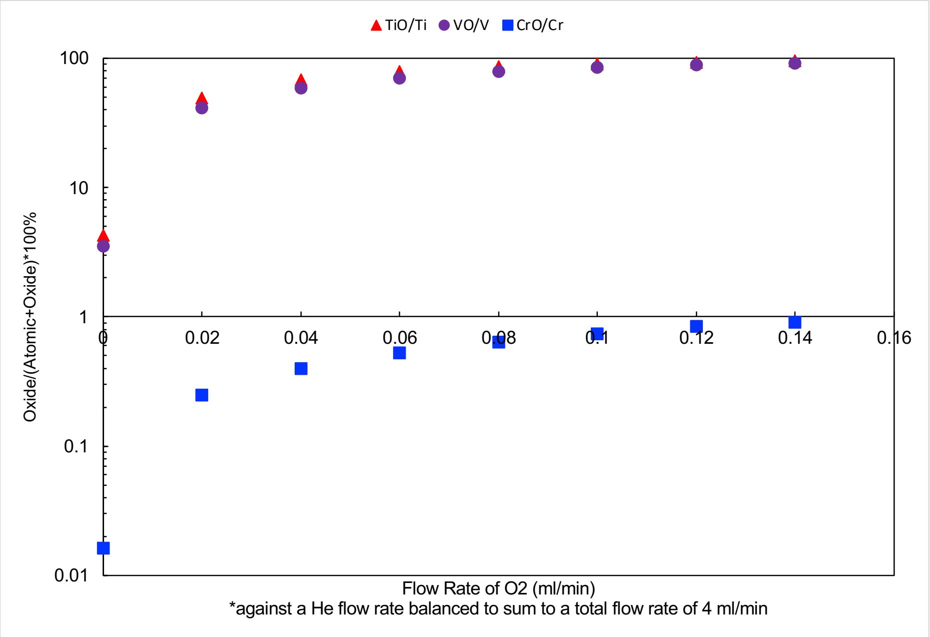

Supplementary figure 5: a) Conversion ratios of oxide to metal for Ti, V and Cr for stepped increased in flow rates of O_2 (actually a He-O_2 95:5 mix) gas and additional He gas, He flow rate always 10x the O_2. b) Conversion ratios of oxide to metal for Ti, V and Cr for different flow rates of O_2 (actually a He-O_2 95:5 mix) gas, with an additional He gas flow rate balanced to give a total gas flow rate of 4 ml/min.
